# Dose-response relationships for environmentally mediated infectious disease transmission models

**DOI:** 10.1371/journal.pcbi.1005481

**Published:** 2017-04-07

**Authors:** Andrew F. Brouwer, Mark H. Weir, Marisa C. Eisenberg, Rafael Meza, Joseph N. S. Eisenberg

**Affiliations:** 1 Department of Epidemiology, University of Michigan, Ann Arbor, MI, United States of America; 2 Division of Environmental Health Sciences, The Ohio State University, Columbus, OH, United States of America; University of California, Los Angeles, UNITED STATES

## Abstract

Environmentally mediated infectious disease transmission models provide a mechanistic approach to examining environmental interventions for outbreaks, such as water treatment or surface decontamination. The shift from the classical SIR framework to one incorporating the environment requires codifying the relationship between exposure to environmental pathogens and infection, i.e. the dose–response relationship. Much of the work characterizing the functional forms of dose–response relationships has used statistical fit to experimental data. However, there has been little research examining the consequences of the choice of functional form in the context of transmission dynamics. To this end, we identify four properties of dose–response functions that should be considered when selecting a functional form: low-dose linearity, scalability, concavity, and whether it is a single-hit model. We find that i) middle- and high-dose data do not constrain the low-dose response, and different dose–response forms that are equally plausible given the data can lead to significant differences in simulated outbreak dynamics; ii) the choice of how to aggregate continuous exposure into discrete doses can impact the modeled force of infection; iii) low-dose linear, concave functions allow the basic reproduction number to control global dynamics; and iv) identifiability analysis offers a way to manage multiple sources of uncertainty and leverage environmental monitoring to make inference about infectivity. By applying an environmentally mediated infectious disease model to the 1993 Milwaukee *Cryptosporidium* outbreak, we demonstrate that environmental monitoring allows for inference regarding the infectivity of the pathogen and thus improves our ability to identify outbreak characteristics such as pathogen strain.

## Author summary

Many infectious disease interventions, including water treatment, hand hygiene, and surface decontamination, target pathogens in the environment. Explicitly modeling the concentration of pathogens in the environment within transmission models can be a useful way to consider not only the impact of such mitigation efforts but also the spatial spread of pathogens and sampling strategies for environmental monitoring. However, we need to understand the dose–response relationship, that is, how exposure to pathogens translates into a probability of infection. The field of quantitative microbial risk assessment has developed dose–response models from experimental data, but little work has been done to assess the impact the choice of dose–response model has on transmission model dynamics. We show that dynamics of simulated transmission models incorporating a dose–response model that has been fit to experimental data can vary widely despite little statistical difference in the fit to the experimental dose–response data. This and other results allow us to give specific guidance for the use of dose–response functions in a transmission modeling context. We also underscore the usefulness of environmentally mediated transmission models by demonstrating how environmental monitoring data can be used to provide new information about pathogen strain.

## Introduction

Modeling infectious disease transmission by person-to-person contact has a long history in the scientific community. Environmentally mediated transmission modeling, on the other hand, has only recently been explored, notably including i) the Codeço model [1] (based on an older model by Capasso and Paveri-Fontana [2]) that has strongly influenced the field of cholera modeling (e.g. [3–9]); ii) a series of enteric pathogen transmission models that investigated multiple transmission pathways [10–13]; iii) the Environmental Infection Transmission System (EITS) model [14], which suggested that properties of the environment could mediate between frequency- and density-dependent transmission; and iv) the SIWR (Susceptible, Infectious, Water, Recovered) model [15–19], which has been used to develop analytic results for models of waterborne disease with multiple transmission pathways, especially on networks.

Explicit modeling of pathogens in the environment can generate additional insight into how environmental processes affect infectious disease dynamics and allow modelers to incorporate knowledge from experimental studies into their models. In particular, it allows for consideration of pathogen fate and transport [20] and the functional relationship, called the dose–response relationship, between the amount of pathogen a person is exposed to (dose) and the probability of infection, illness, or death (response). Ultimately, because many interventions work through environmental media (e.g. water treatment, surface decontamination, etc.), environmental modeling can improve the predictions arising from disease transmission models, enhancing its applicability to outbreak control and mitigation planning.

Microbial dose–response modeling has largely grown out of the field of quantitative microbial risk assessment (QMRA) and was established, for gastrointestinal pathogens in particular, by seminal work by Haas [21, 22] and Teunis [23]. Work in this area has emphasized the biological plausibility of the exponential and beta–Poisson single-hit models, which semi-mechanistically model pathogen distribution in doses and survival in the host. Empirical models, which come from the field of chemical toxicology and are based on the theory of tolerance distributions [24], have also been used, particularly for foodborne diseases [25, 26]. In practice, those seeking to develop a dose–response relationship for QMRA must find data for an appropriate host organism that aligns with the exposure route and desired endpoint (e.g. symptoms or clinical infection) [27]. Once appropriate data are found, the choice of functional form from among a plausible set is usually a statistical one (goodness-of-fit or best-fit).

In applied work, transmission modelers have thus far considered only the most mathematically tractable of dose–response relationships in transmission models that explicitly consider pathogen dynamics: the Codeço model uses the Hill-1 function and both the EITS and SIWR model use a linear relationship between pathogen levels in the environment and the probability of infection. However, the relationship between exposure and infection risk could be more complex, and the consequences of the misspecification of this relationship on model dynamics and predictions have not been explored in detail. Theoretical work, such as by Wang and Liao [28], has generally been agnostic to the functional form of the dose–response relationships and does not compare the forms used in experimental science or consider which are most appropriate for practical applications. Thus, there remains a clear need to formalize which dose–response forms should be used in transmission modeling and what considerations are necessary for evaluating competing dose–response models. To address this need, here we

1. examine the extent to which dose–response data constrains the dose–response functional forms and the dynamics of a disease transmission model,
2. address potential problems that arise when continuous exposure is aggregated in discrete doses through the specification of environmental contact and pathogen pick-up rates,
3. consider the properties of common dose–response functions and prove that concave, low-dose linear dose–response behaviors are needed to ensure that global model dynamics remain controlled by the familiar basic reproduction number $R_{0}$, and
4. use identifiability analysis to demonstrate that the impact of the choice of dose–response function can be mitigated in many circumstances.

Finally, we apply our methodology to the 1993 Milwaukee *Cryptosporidium* outbreak, using the identifiability results to demonstrate the added power of environmental monitoring.

## Models and methods

### Dose–response functions

A dose–response function *f* connects a number of pathogens (dose *x*), with a probability of infection (response *f*(*x*)). Biologically justified dose–response functions have the following properties: i) zero probability of infection when there are no pathogens (*f*(0) = 0); ii) larger probabilities of infection at larger doses (*f* is increasing); and iii) saturation at 100% probability of infection for an infinite dose (lim_*x* → ∞_ *f*(*x*) = 1). Hence, any cumulative distribution function can, in principle, be a dose–response function. Here, we consider eight dose–response functions that are used in QMRA, mathematical modeling, or experimental science: the linear, exponential, exact beta–Poisson, approximate beta–Poisson (also known as Lomax), Hill-1 (also known as Michaelis–Menten or Langmuir), Hill-*n* (also known as log-logistic), log-normal (also known as log-probit), and Weibull functions. The equations and selected properties for these functions are given in Table 1. Although several other empirical functions are also fit to dose–response data, we restrict ourselves to the most common examples.

**Table 1:** Common dose–response functions and selected properties. Equations give a probability of infection *f*(*x*) (response) for a number of pathogens *x* (dose). Parameter *π* = *f*′(0) for the functions with finite, non-zero slope at the origin. Some functions have been given non-standard parameterizations in order facilitate comparison between functions. In particular, the beta–Poisson functions are often parameterized in terms of *α* = *πβ* and *N*_50_ = *β*(2^1/*α*^ − 1). Some functions are known by different names with different parameterizations. Hill-*n* is called log-logistic when written in terms of *β* = −*n* and *α* = 1/*π*, and log-normal is called log-probit when written in terms of *β*_0_ = −*μ*/*σ* and *β*_1_ = 1/*σ*.

Model	Equation *f*(*x*)	Single-hit	Low-dose linear^a^	Scalable	Concave
Linear	$\{\begin{array}{cc} \pi x & x\leq \frac{1}{\pi }\\ 1 & x>\pi \end{array}$	No	Yes	Yes	Yes
Exponential	1 − *e*^−*πx*^	Yes	Yes	Yes	Yes
Exact beta–Poisson^b^	1 − _1_*F*_1_(*πβ*, *β*(*π* + 1), − *x*)	Yes	Yes	No	Yes
Approximate beta–Poisson	$1-{\left(1+\frac{x}{\beta }\right)}^{-\pi \beta }$	Yes^c^	Yes	Yes	Yes
Hill-1	$\frac{x}{\frac{1}{\pi }+x}$	Yes^c^	Yes	Yes	Yes
Hill-*n*	$\frac{x^{n}}{{\left(\frac{1}{\pi }\right)}^{n}+x^{n}}$	No	No	Yes	Depends^d^
Log-normal^e^	$\Phi \;(\frac{\ln x-\mu }{\sigma })$	No	No	Yes	No
Weibull	1 − *e*^−(*πx*)^*n*^^	No	No	Yes	Depends^d^

^a^: Finite, non-zero low-dose linear.

^b^: _1_*F*_1_(*a*, *b*, *x*) is the confluent hypergeometric function of the first kind.

^c^: With negative-binomial rather than Poisson distribution of organisms; see text.

^d^: Depends on the value of the parameters. Both Hill-*n* and Weibull are concave for *n* ≤ 1.

^e^: Writing the log-normal function in integral form demonstrates that it is well-defined at *x* = 0.

We consider four properties of these dose–response functions, which we subsequently define in greater detail: i) whether they are single-hit models, ii) whether they low-dose linear, iii) whether they are scalable, and iv) whether they are concave. *Single-hit* models are considered by many to be the most biologically plausible of the common dose–response relationships [29] and are derived from two main assumptions, namely that a single organism can cause an infection and that pathogens act independently [30]. We discuss the form and derivation of single-hit models in greater detail below.

The behavior of dose–response functions in the low dose regime, not only for pathogens but also for radiation, chemical exposure, etc., is difficult to assess experimentally. Several theoretical behaviors have been posited, including thresholding (no effect below a certain exposure), linearity (effect proportional to dose even at the lowest doses), and hormesis (small exposures have a net benefit). Arguments have been made for low-dose linearity for radiation and chemical exposures [31, 32], and for pathogens, experimental data are not consistent with thresholds: threshold models have a steeper slope at the median dose than the exponential model, but nearly all experimental data indicate slopes equal to, or shallower than, the exponential model [29]. Moreover, it is generally well-accepted that a single organism is capable of causing disease [29]. Here, we distinguish *low-dose linear* model behavior by the technical property 0 < *f*′(0) < ∞.

A dose–response model is *scalable* if there is a parameterization of the function in which the dose *x* and some parameter appear only as a product with the other. If a model is scalable, the shape of the dose–response function will not depend on the units of the dose. Scalability is most important when measurements of pathogen concentration are not necessarily in units of individually infectious organisms, which is particularly common for viruses, where doses may be measured in TCID_50_ (median tissue culture infectious dose), pfu (plaque forming units), or ffu (focus-forming units), etc., rather than number of individual viruses.

Convexity (*f*″(*x*) ≥ 0) in a dose–response function means that an additional pathogen in a dose increases the probability of infection beyond what it would independently and implies synergy, or cooperation, between pathogens. Pathogen independence, on the other hand, assumes no cooperation and implies *concavity* (*f*″(*x*) ≤ 0), due to saturation effects. Review of experimental evidence points toward independent action [33], and, thus, argues for the use of concave dose–response functions. Please note that functions that are concave may not appear so when plotted with dose on the log-scale.

#### Single-hit models

Single-hit models are derived from assumptions about two processes, one environmental—an individual must be exposed to at least one pathogen—and one within-host—each pathogen has an independent probability of surviving to cause infection [29]. Single-hit models can be written in the form

$$P\left(x\right)=\sum_{k=1}^{\infty }\sum_{j=k}^{\infty }\;P_{1}\left(j|x\right)P_{2}\left(k|j\right)$$ (1)

where *P*_1_(*j*|*x*) is the probability of being exposed to *j* pathogens when the mean dose is *x* and *P*_2_(*k*|*j*) is the probability that *k* out of *j* organisms survive the exposure event and go on to cause an infection [29]. An extensive mathematical consideration of single-hit functions may be found elsewhere [30, 34]. The model for *P*_1_ is most usually taken to be Poisson, which assumes that pathogens are randomly distributed in the environment. Given that pathogens are often clustered, the negative-binomial distribution, which has larger-than-Poisson variance, is also used [34–36].

Four of the dose–response functions we are considering are technically single-hit models. The exponential function assumes a Poisson-distributed number of pathogens in an exposure and assumes that all pathogens have the same independent probability of surviving in the host to cause infection (i.e. *P*_2_ is binomial). The exact beta–Poisson function similarly uses a Poisson-distributed dose but allows variability in the probability that pathogens survive to cause an infection. This variability is accomplished using the beta distribution, which is very flexible but does not have mechanistic underpinnings. Although the beta–Poisson function is typically parameterized with the parameters of the beta distribution (*β* and *α*), in this study we use a non-standard parameterization (*β* and *π* = *α*/*β*) in order to facilitate comparison of the low-dose slope of the different dose–response functions. The approximate beta–Poisson function is often used in lieu of the exact beta–Poisson model. It is not a single-hit model if we insist upon Poisson-distributed pathogens in the environment [30, 37]; however, the approximate beta–Poisson function can be considered a single-hit model with a constrained negative–binomial distribution of pathogens and a constant infection probability. This interpretation requires an assumption, namely that the negative-binomial clustering parameter depends on the dose. This assumption has been considered implausible [30] and, thus, should be used with caution. The Hill-1 function, as a simplification of the approximate beta–Poisson (*πβ* = 1), is thus also a single-hit model, though the same caveat applies.

From a biological perspective, the exact beta–Poisson model is perhaps the most realistic dose–response function. However, its functional form uses a confluent hypergeometric function, which is not only difficult to work with but can become numerically intractable for large values of *β*. Where direct calculation of the exact beta-Poisson function is not possible, we use a numerical solution to Kummer’s equation,

$$x\frac{d^{2}y}{dx^{2}}+\left(b-x\right)\frac{dy}{dx}-ay=0.$$ (2)

Here, *y* = _1_*F*_1_(*a*, *b*, *x*) may be computed by solving Eq (2) for the value of *y* at dose *x* with initial condition *x* = 0 (in practice, computer precision is used to avoid dividing by 0), *y* = 1, *dy*/*dx* = *a*/*b* [29].

In practice, QMRA modelers typically use the approximate beta–Poisson function, which was derived by Furumoto and Mickey [38, 39]. The approximation is valid when 1 ≪ *β* and *π* ≪ 1, though the parameter ranges for which the approximation is acceptable are not known generally [37]. Schmidt et al. [40] proposed criteria for accepting the approximation.

Several authors have compared the exact and approximate beta–Poisson functions’ fit to data for different pathogens and have reported mixed results with respect to their comparability [29, 37]. Because the exact beta–Poisson function is not scalable (that is, its shape depends on the units of the dose), it should only be applied when there is confidence that the presented dose units are on the scale of single infectious pathogens (as is required by its derivation). It is difficult to confirm that this is the case when the dose is given in units of ffu or TCID_50_, i.e. although these quantitative units may represent some (generally unknown) number of individual infective particles, it is unlikely that they are equal to the number of infective particles. Hence, disagreements between the exact and approximate beta-Poisson formulations may be an artifact of the scale of the dose. We will not present exact beta–Poisson dose–response fits when the scale is in question.

#### Empirical models

Empirical dose–response functions use functional forms that do not currently have a plausible biological basis, though many are based on the theory of tolerance distributions, which grew out of chemical toxicology [24, 29]. A susceptible population is assumed to have an inherent tolerance distribution, and individuals are only infected if the dose exceeds their tolerance level. The three empirical relationships that we consider—namely the log-logistic, log-normal, and Weibull—are named for the tolerance distributions they assume.

### Disease transmission model

**Fig 1:**
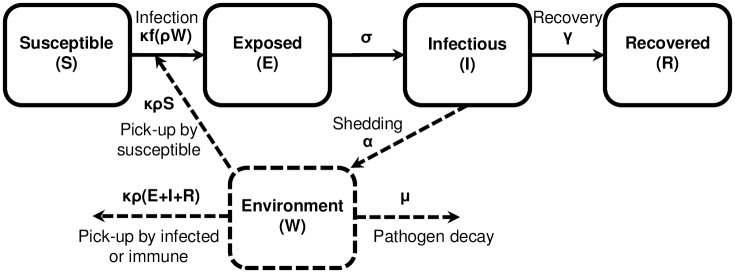
Schematic of an environmentally mediated infectious disease transmission model with dose–response and exposed compartment. Solid lines represent people and dashed lines represent pathogens. Variables and parameters are defined in Table 2.

We consider an environmentally mediated infectious disease transmission model—based on the EITS and SIWR models [14, 15]—that includes a dose–response relationship and a latent period. All pathogens we consider are more realistically modeled with a latent period, incorporated by including one or more compartments for an exposed class of individuals who have become infected but are not yet infectious. Although one compartment is often sufficient, additional compartments reduce the variance of the modeled latent period (see S1 Appendix for model extensions). A schematic of the model is shown in Fig 1, the variables and parameters of the model are given in Table 2, and the equations are as follows.

$$\begin{array}{ccc} \dot{S} & = & -\kappa f(\rho W)S\\ \dot{E} & = & \kappa f(\rho W)S-\sigma E\\ \dot{I} & = & \sigma E-\gamma I\\ \dot{R} & = & \gamma I\\ \dot{W} & = & \alpha I-\xi W \end{array}$$ (3)

Although the original EITS model counted the number of pathogens in the environment, it is more straightforward for our purposes to track the concentration of pathogens. We also parameterize the sum of pathogen pathogen pick-up *κ*(*ρ*/*V*)*N* and pathogen decay *μ* rates as a single pathogen removal rate parameter *ξ* = *κ*(*ρ*/*V*)*N* + *μ*. The corresponding model tracking numbers of pathogens may be found in the supplment.

**Table 2:** Model variables and parameters. Compartments and parameterizations of an environmentally mediated infectious disease transmission model with dose–response and a latency period, Eq (3).

**Variables**	
*S*(*t*)	Number of susceptible people
*E*(*t*)	Number of exposed people
*I*(*t*)	Number of infectious people
*R*(*t*)	Number of recovered people
*W*(*t*)	Concentration of pathogens in the environment
**Parameters**	
*γ*	Recovery rate (per day)
*κ*	Rate at which individuals contact the environment (per day)
*ρ*	Volume of environment consumed (per contact)
*V*	Total volume of the environment
*α*	Deposition rate of pathogens per unit volume of environment (per day)
*μ*	Pathogen decay rate (per day)
*ξ*	Removal rate of pathogens; sum decay and pick-up rates (per day)
*σ*	Rate of moving from a latent to infectious state (per day)
**Functions**	
*f*(⋅)	Dose–response function; probability of infection given a dose

We also analyze the corresponding model with a linear dose–response relationship, where a linear pathogen infectivity parameter *π*, which corresponds to the low-dose linear slope *π* of the functions given in Table 1, replaces the dose–response function.

$$\begin{array}{ccc} \dot{S} & = & -\pi \kappa \rho WS\\ \dot{E} & = & \pi \kappa \rho WS-\sigma E\\ \dot{I} & = & \sigma E-\gamma I\\ \dot{R} & = & \gamma I\\ \dot{W} & = & \alpha I-\xi W \end{array}$$ (4)

The models in Eqs (3) and (4) may differ from other environmentally mediated transmission models in a number of ways, including that we do not consider human birth and death (that is, we have a constant population *N* = *S* + *E* + *I* + *R*) as we are considering epidemic, rather than endemic, timescales, and we do not include a distinct person-to-person transmission pathway. While variations to these assumptions are important in some contexts, we focus here on a simple model to highlight the impact of including a dose–response relationship on environmentally mediated transmission. Equations for versions of the model incorporating person-to-person transmission or birth–death dynamics are included in S1 Appendix for reference, and we discuss the robustness of the basic reproduction number results to changes in these assumptions in a later section.

#### The basic reproduction number

A key concept in infectious disease epidemiology is the basic reproduction number $R_{0}$, defined as the average number of secondary cases arising from a typical primary case in an entirely susceptible population. In practice, the basic reproduction number is used for its epidemic-threshold, that is *local stability*, properties: for initial conditions sufficiently near the disease-free equilibrium, if $R_{0}<1$, the disease will die-off, and if $R_{0}>1$, then there will be an epidemic.

For ordinary differential equation (ODE) models, the basic reproduction number is often calculated using the Next Generation Method. Although detailed at length elsewhere [41, 42], we provide a sketch of the method to facilitate understanding of the later proofs. Denote the vector of infected states by *x*, the uninfected states by *y*, and the disease free equilibrium by (*x*_0_, *y*_0_). For each infected compartment *i*, let $F_{i}\left(x,y\right)$ denote the rate at which previously susceptible individuals enter compartment *i* and $V_{i}\left(x,y\right)$ denote the net rate of transfer of individuals out of compartment *i*. Then

$$\dot{x}=F-V.$$ (5)

Denote by *F* and *V* the matrices whose entries are

$$F_{ij}=\frac{\partial F_{i}}{\partial x_{j}}{|}_{\left(x_{0},y_{0}\right)}$$ (6)

$$V_{ij}=\frac{\partial V_{i}}{\partial x_{j}}{|}_{\left(x_{0},y_{0}\right)}$$ (7)

Then, the matrix *K* = *FV*^−1^ is called the next generation matrix. Its (*i*, *j*)th entry is the expected number of new infections in compartment *i* produced by an individual introduced to compartment *j*. The basic reproduction number $R_{0}$ is the spectral radius (largest eigenvalue) of *K*.

### Computational methods

#### Maximum-likelihood estimators and confidence intervals for dose–response functions

We fit dose–response models to experimental data using maximum likelihood estimation. In particular, we assume that the number of people infected by dose *x*_*i*_ follows a binomial distribution with probability *f*(*x*_*i*_) and number of subjects *n*_*i*_. Then, the likelihood, as a function of the dose–response parameters *θ*, is given by

$$L\left(\theta \right)=\prod_{i}\;\frac{k_{i}!}{k_{i}!\left(n_{i}-k_{i}\right)!}{\left(f(x_{i},\theta )\right)}^{k_{i}}\;{\left(1-f(x_{i},\theta )\right)}^{n_{i}-k_{i}},$$ (8)

where *k*_*i*_ is the number of infected people at dose *x*_*i*_. We minimized the negative log-likelihood (NLL) using the NMinimize function in Mathematica 10.2.

We calculated confidence intervals for the dose–response functions by first approximating the likelihood-based 95% confidence region of parameter space. This region is defined as all parameter vectors that have a NLL within

$$\Delta =\chi^{2}\left(0.05,\text{df}\right)/2$$ (9)

of the minimal NLL, where *χ*^2^(0.05, df) is the *χ*^2^ distribution with level of significance 0.05 and degrees of freedom equal to the number of parameters in the dose–response function [43]. Because the dose–response functions are continuous, this region is easily found for single parameter functions by direct numerical computation. Regions for multiparameter dose–response functions are more difficult to approximate, and we used a Markov-chain Monte Carlo (MCMC) approach in the following way. First, we estimated a covariance matrix for the dose–response parameters by generating a Markov-chain with the NLL function (Eq (8)) in the mymcmc function in the Bhat package in R (v3.3.1) [44], burning 1,000 and keeping 10,000. We then generated 100,000 samples from a multivariate normal distribution with the mean parameter values found from the maximum likelihood estimation above and the covariance matrix estimated from the Markov chain. We then approximated the likelihood-based confidence region by taking the subset of samples that had a NLL within the designated range. We calculated the dose–response function for each sample and define the 95% confidence interval for the maximum likelihood dose–response function at each dose to be the range of response values over all parameter vectors in the confidence region.

#### Transmission model simulation and parameter estimation

Integration of the ODE systems given in Eqs (3) and (4) was done using the ode function in the deSolve package in R. Parameter estimation for these models from case or environmental monitoring data was also accomplished using maximum likelihood estimation. Case data *K*_*i*_ were assumed to be binomially distributed with a probability of infection given by the fraction of infected people *I*(*t*_*i*_, *ψ*)/*N* in the ODE model with parameters *ψ* at time *t*_*i*_, that is

$$L\left(\psi \right)=\prod_{i}\;\frac{K_{i}!}{K_{i}!\cdot \left(N-K_{i}\right)!}{\left(I(t_{i},\psi )/N\right)}^{K_{i}}\;{\left(1-I(t_{i},\psi )/N)\right)}^{N-K_{i}}.$$ (10)

The number of pathogens *Q*_*i*_ in an environmental sample were assumed to be Poisson distributed with mean *W*(*t*_*i*_, *ψ*) ⋅ *v*_*i*_, where *v*_*i*_ is the sample volume and *W*(*t*_*i*_, *ψ*) is the environmental pathogen concentration given by the ODE model—again with parameters *ψ*—at the corresponding time, that is,

$$L\left(\psi \right)=\prod_{i}\;\frac{{\left(W\left(t_{i},\psi \right)\cdot v_{i}\right)}^{Q_{i}}\;e^{-W\left(t_{i},\psi \right)\cdot v_{i}}}{Q_{i}!}.$$ (11)

We minimized the negative log-likelihood for the transmission models using the David-Fletcher-Powell optimization algorithm (dfp) in the Bhat package in R.

## Results and discussion

### Dose–response functions fit to data: Implications for dynamics

**Fig 2:**
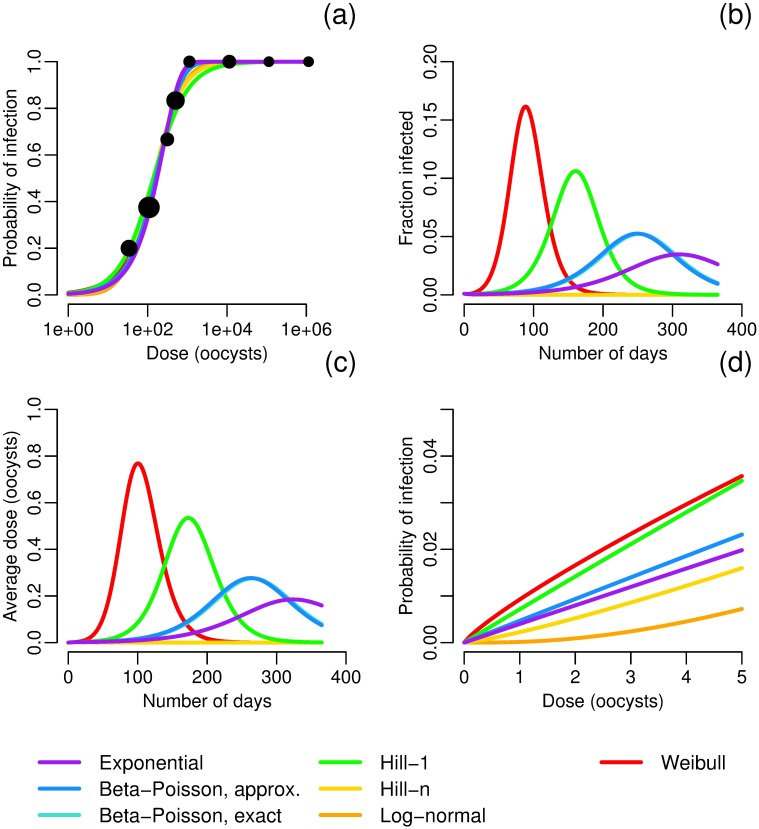
*Cryptosporidium* dose–response and dynamics. a) Maximum-likelihood estimates of dose–response functions for *Cryptosporidium*. Data from [45]; sizes of data points correspond to sample size. Best-fit parameters are given in Table 3. b) Modeled fraction of infected people under different dose–response relationships. Model parameters are *N* = 1000, *S*_0_ = 999, *I*_0_ = 1, *W*_0_ = 0, *σ* = 1/7 [46], *γ* = 1/10 [46], *κ* = 8 and *ρ* = 0.15 so that *κρ* = 1.2 L [47], *V* = 4*E*8 L, *α* = 1E6/*V* (taken from a range [48]), *μ* = 0.069 (taken from a range [49]). Model results using the exact and approximate beta–Poisson functions lie nearly on top of each other, and those using the Hill-*n* and log-normal functions have no outbreak. c) Average pathogen dose over time for different dose–response relationships. Model parameters are as in Fig 2b. d) Low dose behavior of the dose–response functions given in Fig 2a. Confidence intervals are given in Fig 3.

Here we present a number of examples to highlight the impact the choice of a dose–response function has on model dynamics. As an initial example, we fit seven dose–response functions to data for the Iowa strain of *Cryptosporidium parvum* [45] by maximum likelihood estimation. Best-fit parameters and negative log-likelihoods are given in Table 3. There is good agreement, qualitatively, among the seven functions (Fig 2a); in particular, the exact and approximate beta–Poisson models are indistinguishable. These seven functions are used as the dose–response relationship *f* in the model given in Eq (3), parameterized to reasonably approximate *Cryptosporidium* (note that parameters with significant uncertainty, *V* and *α* in particular, were chosen so that the exponential model and beta–Poisson models, which are the models most commonly used in practice, give reasonable outbreaks). Despite the seeming agreement among the dose–response functions, the corresponding dynamics differ significantly in the total size of the outbreak and the timing and size of the outbreak peak (Fig 2b, Table 4). The differences in dynamics are solely a result of the shape of the dose–response function; each simulation uses the same model, parameters, and initial conditions and differs only in the choice of dose–response function. The following observations help to understand this phenomenon. First, the dynamics of these simulations are driven by low-dose exposure: the average number of pathogens per exposure does not exceed one pathogen for any simulation at any time (Fig 2c). Second, when we consider only the low-dose range of the dose response functions (Fig 2d), there is a substantial spread in the dose–response behavior, resulting in markedly different reproductions numbers (Table 4).

**Table 3:** Best fit parameters and negative log-likelihood (in parantheses) for the pathogens and dose–response functions given in Figs 2, 4 and 5. The negative log-likelihood that admits the best fit by the Akaike information criterion is in bold. A table including results for influenza, rotavirus, and *Salmonella typhi* may be found in S1 Appendix.

Pathogen	Exponential	Exact beta–Poisson	Approximate beta–Poisson	Hill-1	Hill-*n*	Log-normal	Weibull	Reference
*Cryptosporidium parvum*	*π* = 4.005E-3	*π* = 4.718E-3,	*π* = 4.702E-3	*π* = 7.187E-3	*π* = 6.985E-3	*μ* = 4.954*E*1	*π* = 4.010E-3	[45]
		*β* = 9.046E2	*β* = 9.042E2		*n* = 1.229E0	*σ* = 1.367*E*0	*k* = 8.478E-1	
	(**12.5872**)	(12.5514)	(12.5513)	(12.7919)	(12.6777)	(12.6487)	(12.4922)	
*Shigella flexneri*	*π* = 6.837E-7	*π* = 1.141E-2	*π* = 1.188E-2	*π* = 1.333E-4	*π* = 2.446E-4	*μ* = 8.324E0	*π* = 8.630E-6	[52, 53]
		*β* = 1.027E1	*β* = 9.864E0		*n* = 1.869E-1	*σ* = 8.903E0	*k* = 1.083E-1	
	(666.769)	(**154.937**)	(154.939)	(221.665)	(156.034)	(156.123)	(156.672)	
*Vibrio cholerae*, buffered	*π* = 8.490E-6	*π* = 1.5463E-2	*π* = 1.542E-2	*π* = 3.644E-4	*π* = 5.641E-3	*μ* = 4.909E0	*π* = 1.627E-3	[50]
		*β* = 1.610E1	*β* = 1.620E1		*n* = 3.207E-1	*σ* = 5.721E0	*k* = 1.372E-1	
	(63.8322)	(**18.2152**)	(18.2198)	(25.6891)	(18.7615)	(18.9054)	(19.2063)	

**Table 4:** Attack ratios and basic reproduction numbers $R_{0}$ by dose–response function. The attack ratio, or cumulative incidence, is the fraction of at-risk (susceptible) individuals in the population who become infected during the outbreak. The simulations of a *Cryptosporidium* outbreak are pictured in Fig 2b. Because some transmission model parameters are uncertain, these values are for comparison between functions only and do not necessarily reflect real-world dynamics.

Dose–response function	Attack ratio	$R_{0}$
Exponential	0.75	1.9
Exact beta–Poisson	0.84	2.2
Approximate beta–Poisson	0.84	2.2
Hill-1	0.96	3.3
Hill-*n*	0.00	0.0
Log-normal	0.00	0.0
Weibull	0.99	∞

**Fig 3:**
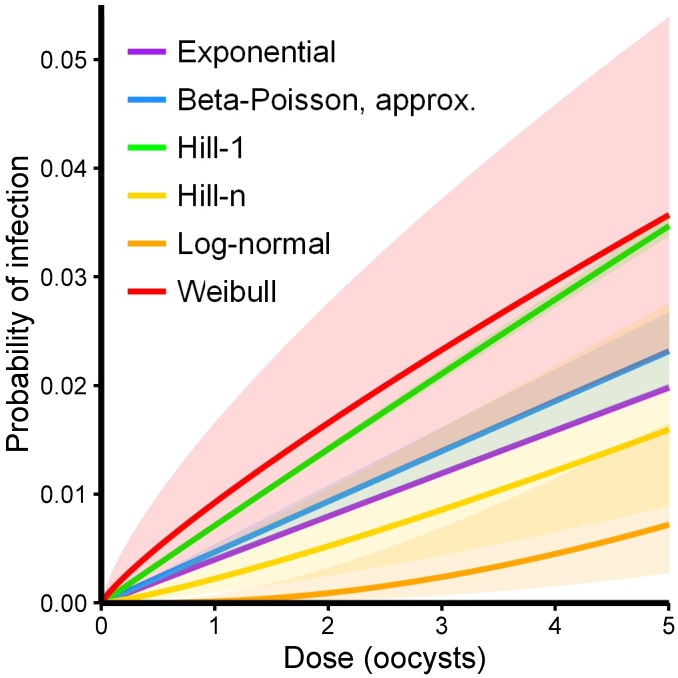
Low-dose regime of the *Cryptosporidium* dose–response functions (see Fig 2d) with 95% confidence intervals determined by likelihood-based confidence regions for the underlying parameters.

Choosing a dose–response functional form based on statistical fit alone is problematic; the probabilities of infection in the low-dose range, which control the dynamics, differ widely over the possible functional forms despite nearly equivalent statistical fits to the experimental data. There is much less uncertainty in the low-dose regime for any one functional form (especially the one-parameter forms) than is seen across the gamut of functional forms (Fig 3), and thus the choice of a functional form by statistical test can artificially shrink the confidence bounds of the results. Hence, it is important for modelers to conduct sensitivity analyses when selecting a dose–response function.

To further explore the issue incorporating dose–response models into transmission models, we next consider dose–response functions and corresponding dynamics for *Vibrio cholerae* and *Shigella flexneri*. Analogous analyses for influenza, rotavirus, and *Salmonella typhi* may be found in S1 Appendix.

**Fig 4:**
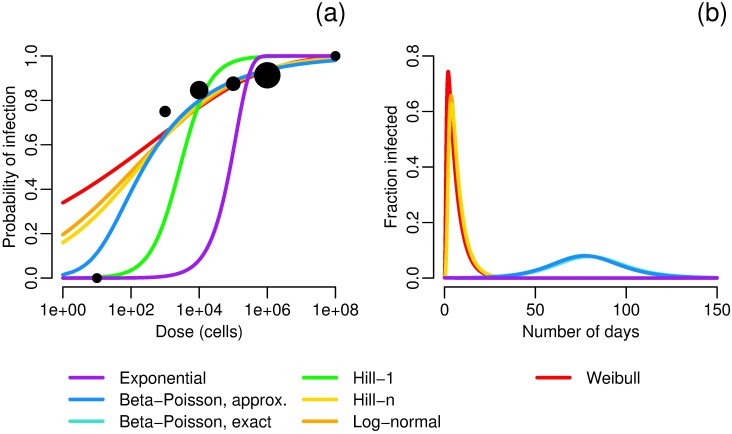
*Vibrio cholerae* dose–response and dynamics. a) Maximum-likelihood estimates of dose–response functions for buffered Inaba strain of *Vibrio cholerae*. Buffering was used in this experiment to approximate eating contaminated food, as food buffers stomach acid; because *Vibrio cholerae* does not tolerate gastric acidity, a buffered strain is more infectious. Data from [50]; sizes of data points correspond to sample size. Best-fit parameters are given in Table 3. b) Modeled fraction of infected people under different *Vibrio cholerae* dose–response relationships. Model parameters are *N* = 1000, *S*_0_ = 999, *I*_0_ = 1, *W*_0_ = 0, *σ* = 5/2 [51], *γ* = 1/5 [9], *κ* = 8 and *ρ* = 0.15 so that *κρ* = 1.2 L [47], *V* = 4*E*8, *α* = 2E6/*V*, *μ* = 0.23 [9]. Model results using the exact and approximate beta–Poisson functions lie nearly on top of each other, and those using the Hill-1 and exponential functions have no outbreak.

**Fig 5:**
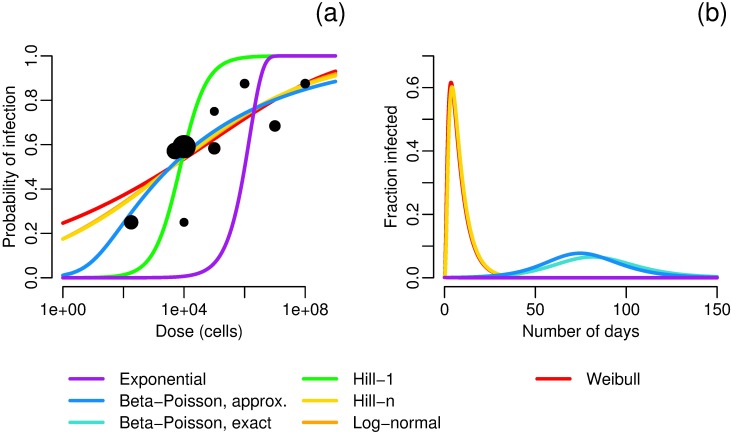
*Shigella flexneri* dose–response and dynamics. a) Maximum-likelihood estimates of dose–response functions for *Shigella flexneri*. Data from [52, 53]; sizes of data points correspond to sample size. Best-fit parameters are given in Table 3. b) Modeled fraction of infected people under different *Shigella flexneri* dose–response relationships. Model parameters are *N* = 1000, *S*_0_ = 999, *I*_0_ = 1, *W*_0_ = 0, *σ* = 2/3 [54], *γ* = 1/6 [54], *κ* = 8 and *ρ* = 0.15 so that *κρ* = 1.2 L [47], *V* = 4*E*8, *α* = 4E7/*V*, *μ* = 5 [55, 56]. Model results using the Weibull, log-normal, and Hill-*n* functions lie nearly on top of each other, and those using the Hill-1 and exponential functions have no outbreak.

For *Vibrio cholerae*, there is significantly less agreement among the dose–response functions than there was for *Cryptosporidium* (Fig 4). The Weibull, Hill-*n* and log-normal functions have unrealistically high modeled single-pathogen infection probabilities (around 0.2–0.4). Moreover, for the Weibull and Hill-*n* functions, $R_{0}$ takes the uninterpretable value of ∞ and will have an outbreak for every initial condition. For the log–normal function, $R_{0}=0$; even though $R_{0}<1$, because the log-normal function is convex at the origin and the initial conditions are not sufficiently close to the disease-free equilibrium, this is a scenario in which an outbreak is nonetheless observed. The same is true for *Shigella* (Fig 5).

While the exact and approximate beta–Poisson models gave equivalent outbreaks for *Vibrio cholerae*, there is a slight difference in their corresponding outbreak dynamics for *Shigella*, even though there is no visible discrepancy (on this scale) in the dose–response functions. The estimated value of *β* is higher for *Vibrio cholerae* than for *Shigella* (*β* ≈ 16 vs. 10), and, more importantly, the difference between the low-dose slope for the exact and approximate functions is an order of less for *Vibrio cholerae* than for *Shigella* (*π*_aBP_ − *π*_eBP_ ≈ 4E-5 vs. 5E-4). Our results suggest that the approximate beta–Poisson function is indeed an acceptable approximation in most cases but that care should be taken to consider the possibility of discrepancy.

In summary, middle- and high-dose data for fitting dose–response functions do not satisfactorily constrain the behavior of the dose–response model at low doses. Transmission dynamics, especially in non-outbreak conditions, are likely characterized by low-dose conditions [29, 57], and, indeed, our results show that typical outbreak curves can result from low-dose conditions. The choice of an appropriate function for a transmission model, therefore, should not be solely based on either statistical fit or simple mathematical tractability, as misspecification at this level will propagate through the model. By rejecting the empirical functional forms, we can constrain the uncertainty in the low-dose response to some degree, and, in a later section, we demonstrate how we can better manage this uncertainty with identifiability analysis.

There are many sources of uncertainty beyond the choice of dose–response function fit to experimental data. For example, the experimental data is generally obtained from healthy members of the population—or a surrogate host population—using attenuated pathogen strains. Uncertainty, therefore, is introduced because the data may not adequately represent the infection probabilities for a given outbreak. Further, there is uncertainty in several other transmission model parameters, including contact rate and shedding rate, which we discuss in greater detail in upcoming sections.

### Aggregation of exposure into discrete doses: Implications for risk estimation

One unresolved issue for environmentally mediated infectious disease transmission models is how to aggregate environmental exposure into discrete doses correctly. Consider airborne pathogens, for which contact with the environment is the act of breathing. It is difficult to define the contact rate in a meaningful way: Does one breath constitute an independent dose? Or is it one hour of exposure, or one day, that is aggregated to an independent dose? Although enteric pathogens may seem simpler at first (one can define contact as a specific act of ingesting food or water or of touching a contaminated surface), we face the same problem: is a dose a swallow, a glass of water, or all of the water imbibed in a day? For example, if a person ingests 100 infectious cells on three separate occasions in a day, the person’s probability of infection is modeled as 1 − (1 − *f*(100))^3^ if each ingestion event is assumed to be independent, and it is modeled as *f*(300) if we assume that all exposure in a single day can be aggregated into a single dose. In the context of the transmission model, which is focused on the population scale (going from Reed–Frost to Kermack–McKendrick mass-action [58]), the question is whether the force of infection, that is the rate at which susceptible people become infected, is 3*f*(100) or *f*(300).

To use dose–response functions in an environmentally mediated transmission model, we must specify the time-scale on which exposures can be considered independent, that is, we must define the contact rate with environment *κ* (where each contact is an independent exposure) and the per-exposure pathogen pick-up volume *ρ*. In doing so, we must decide whether the total number of pathogens contacted in a day comes in many, small or few, large independent doses. In this section, we show that whether the force of infection is greater or lesser for many, small doses versus few, large doses will depend on the form of the dose–response function.

**Fig 6:**
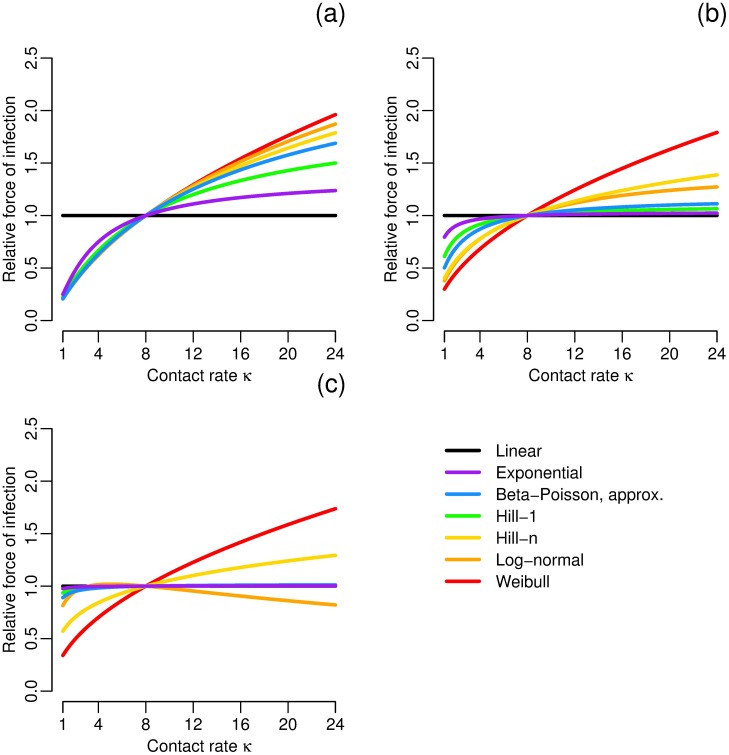
Force of infection changes with dose aggregation. Force of infection *κf*(*ρW*) relative to that when the contact rate is *κ* = 8 for constant daily dose *κρE*, i.e. *κf*(*ρW*)/(8*f*(*κρW*/8)), for each dose–response model parameterized to have an ID_50_ of 1E6 (comparable to influenza) at a) high, b) medium, and c) low total doses. Any parameters not constrained by the median dose were chosen from the best-fit influenza parameters (see S1 Appendix).

To demonstrate the potential effects of how exposure is aggregated on model dynamics, we compare the ingestion of many, small doses with fewer, large doses while keeping the total dose (*κρW*) constant under high, medium, and low total dose conditions. In particular, we consider the daily force of infection *κf*(*ρW*) (assuming *W* is approximately constant on this time scale) relative to the force of infection when *κ* = 8 contacts per day, i.e. we consider *κf*(*ρW*)/(8*f*(*κρW*/8)). The choice of *κ* = 8 here corresponds to independent doses each hour over an eight-hour exposure. [14]. We then vary the contact rate *κ*, keeping the total number of pathogens picked up by an individual in a day (*κρW*) constant (Fig 6). These examples demonstrate the *κ*–*ρ* trade-off across a range of doses.

We find that, for most dose–response functions and doses, increasing the contact rate but keeping the total pathogens picked up constant increases the force of infection. That is, modeling an exposure as more, smaller doses is more likely to cause infection than modeling it as fewer, larger doses. The one exception that we see is for the log-normal function at low doses. In fact, this property is controlled by the concavity/convexity of the function at the reference point, with force of infection increasing with contact rate for concave functions (the response function saturates for low contact rates) and decreasing for convex ones. Further, we see that the *κ*–*ρ* trade-off makes the most difference at higher doses, which may cause misestimation of the dynamics near the peak of the epidemic. Finally, the effects of the *κ*–*ρ* trade-off is minimized, as one would expect, at the lower doses for those functions that are low-dose linear.

The above work assumes that each dose has an independent probability of causing infection, but previous work has demonstrated that dose timing may have an impact on the probability of infection [59, 60]. In fact, Pujol et al. [59] found that the same dose spread over a longer period of time significantly reduced the modeled risk because the explicitly modeled immune system was overwhelmed when the dose was administered in a short time window. That result considers the real, biological effects of dose timing, whereas ours considers model misspecification. It is unclear whether, in practice, the assumption that there is a time threshold below which doses are additive and above which they are independent is realistic. Few experimental studies have included dose-timing in the assessment of exposure effects. One notable example is Brachman et al. [61], in which cynomologus monkeys were exposed to anthrax. These authors and other subsequent analysis [60] found evidence that exposure did not need to accumulate to cause infection.

This analysis suggests that modelers using dose–response functions should carefully consider the implications of their choice of contact and pick-up rates in the context of the pathogen and setting. If aggregated doses stay within the linear range of the dose–response function, then the choice of how doses are aggregated becomes less important. In general, more experimental research is needed to better characterize the impact of dose timing.

### Dose–response functions and the basic reproduction number: Implications for global stability

We first give the basic reproduction number for our environmentally mediated infectious disease transmission model with a dose–response relationship. The mathematics is well-established; indeed the basic reproduction number has been previously calculated for many similar environmentally mediated infectious disease transmission models, e.g. [14, 15, 28, 62, 63]. Nevertheless, we give the proof here because it facilitates the proof of Theorem 1 below.

**Proposition 1.** *The basic reproduction number of the model in* Eq (3) *is*

$$R_{0}=\frac{\alpha \kappa \rho N}{\gamma \xi }\cdot f^{\prime }\left(0\right).$$ (12)

*Proof*. In the notation of the Next Generation Method, let *x* = (*E*, *I*, *W*)^*T*^ be the disease compartments and *y* = (*S*, *R*)^*T*^ the non-disease compartments. Then,

$$F=\left[\begin{array}{c} \kappa f(\rho W)S\\ 0\\ 0 \end{array}\right],$$ (13)

$$V=\left[\begin{array}{c} \sigma E\\ -\sigma E+\gamma I\\ -\alpha I+\xi W \end{array}\right],$$ (14)

and we have new-infection and compartment transfer matrices

$$F=\left[\begin{array}{ccc} \;\;0\;\; & \;\;0\;\; & \kappa \rho Nf^{\prime }\left(0\right)\\ 0 & 0 & 0\\ 0 & 0 & 0 \end{array}\right],$$ (15)

$$V=\left[\begin{array}{ccc} \sigma & 0 & 0\\ -\sigma & \gamma & 0\\ 0 & -\alpha & \;\xi \; \end{array}\right].$$ (16)

Finally,

$$K=FV^{-1}=\left[\begin{array}{ccc} \frac{\alpha \kappa \rho Nf^{\prime }\left(0\right)}{\gamma \xi } & \frac{\alpha \kappa \rho Nf^{\prime }\left(0\right)}{\gamma \xi } & \frac{\kappa \rho Nf^{\prime }\left(0\right)}{\xi }\\ 0 & 0 & 0\\ 0 & 0 & 0 \end{array}\right]$$ (17)

Because the matrix is upper triangular, the spectral radius *ρ*(*K*) is the largest diagonal entry.                                       □

**Remark.** As a consequence of neglecting birth and death rates, the models in Eqs 3 and 4 have not one but many disease-free equilibria. They can be described as {(*S*, 0, 0, *R*, 0): *S* + *R* = *N*}. Above, we have computed $R_{0}$ at the equilibrium with a fully susceptible population, (*N*, 0, 0, 0, 0). However, it is straightforward to write the effective reproductive number R—that is, the average number of secondary cases arising from a typical primary case in a population that is not fully susceptible—when the initial condition is *S*_0_ ≠ *N*:

$$R=\frac{\alpha \kappa \rho S_{0}}{\gamma \xi }\cdot f^{\prime }\left(0\right).$$ (18)

We see that the basic reproduction number is controlled by the derivative of the dose–response function at zero, demonstrating the importance of the low-dose range. The value of *f*′(0) corresponds to the linear infectivity parameter *π* in the formulation of $R_{0}$ in for the original EITS model [14]. However, when replacing *π* by a generic dose–response function *f*, the value of *f*′(0) may cause $R_{0}$ to be zero or infinite, which, although valid from the local-stability perspective, cannot reasonably be interpreted in the sense of the expected number of new infections. Hence, to better facilitate comparison among the dose–response models, we also include the $R_{0}$ for the analogous stochastic model, denoted $R_{0}^{*}$, as derived using branching theory [64]. The proof is left to S1 Appendix. Here, *f*′(0) is replaced by *f*(1).

**Proposition 2.** *The basic reproduction number for the stochastic analog of the model given in* Eq (3) *is*

$$R_{0}^{*}=\cdot \frac{\alpha \kappa \rho N}{\gamma \xi }\cdot f\left(1\right).$$ (19)

Although the basic reproduction number controls the local stability of the disease-free equilibrium for all biologically reasonable models, *global stability* results are much stronger and more useful because they give an epidemic threshold for all initial conditions, not just those sufficiently close to the disease-free equilibrium. When the basic reproduction number controls the global stability of the disease-free equilibrium, counter-intuitive scenarios, such as when an outbreak occurs despite $R_{0}<1$, cannot occur. Here, we prove that concave-down dose–response functions have the desired global stability properties.

As described above, these models do not have a single disease-free equilibrium but rather a set of equilibria with different numbers of susceptible and recovered people summing to *N*. Laukó [65] previously used Lyapunov methods to find criteria that determine the stability of disease-free sets, but the proof we provide here, which we believe will be more intuitive to readers, uses an extension of the method described by Shuai and van den Driessche [66].

**Theorem 1.** *Let* Θ = {(*S*, 0, 0, *R*, 0)^*T*^: *S* + *R* = *N*}, *a one-dimensional subset of the state space* {(*S*, *E*, *I*, *R*, *W*)^*T*^}. *If* *f* *is concave in* Eq (3), *then, if* $R_{0}<1$, Θ *is globally asymptotically stable*.

*Proof*. The aim is to construct a Lyapunov function and thus demonstrate the global asymptotic stability of Θ. First, we note that

$$\begin{array}{c} \Omega =\left\{{\left(S,E,I,R,W\right)}^{T}\;:\;0\leq S\leq N,\;0\leq E+I+R\leq N-S,\;0\leq W\leq \frac{\alpha N}{\xi }\right\} \end{array}$$ (20)

is a compact, invariant set for trajectories of Eq 3. Let *x*, *y*, *F*, and *V* remain as defined in the proof of Proposition 1. Define

$$\begin{array}{ccc} h(x,y) & = & \left(F-V)x-F(x,y)+V(x,y\right)\\ & = & \left[\begin{array}{c} \kappa \rho Wf^{\prime }\left(0\right)N-\kappa f\left(\rho W\right)S\\ 0\\ 0 \end{array}\right]. \end{array}$$ (21)

We will need *h*(*x*, *y*) to be non-negative to construct our Lyapunov function. To this end, assume that *f* is concave. Then *ρWf*′(0) ≥ *f*(*ρW*), and, since *S* ≤ *N*, *h*(*x*, *y*) ≥ 0.

We now construct our Lyapunov function *Q*. Let *ω*^*T*^ be the left eigenvector of *V*^−1^ *F* corresponding to the $R_{0}$ eigenvalue, namely, *ω*^*T*^ = (0, 0, 1). Define

$$\begin{array}{ccc} Q & ≔ & \omega^{T}V^{-1}x\\ & = & \frac{\alpha }{\xi \gamma }\left(E+I\right)+\frac{1}{\xi }W \end{array}$$ (22)

Then, when $R_{0}<1$, *Q* is a Lyapunov function since

$$\dot{Q}=\left(R_{0}-1\right)\omega^{T}x-\omega^{T}V^{-1}h\left(x,y\right)<0,$$ (23)

as *h*(*x*, *y*) is non-negative. Now, $\dot{Q}$ simplifies to

$$\dot{Q}=\frac{\alpha \kappa f(\rho W)}{\xi \gamma }\cdot S-W,$$ (24)

and so $\dot{Q}\equiv 0$ if and only if *W* = 0. Now, Θ is the largest invariant set in {(*S*, *E*, *I*, *R*, *W*)^*T*^: *W* = 0}. Hence by Theorem 2 of Lasalle [67], Θ is globally asymptotically stable.         □

**Remark.** Although the proof was written as if the initial conditions of the system were *S*_0_ = *N*, it is straightforward to see that the proof holds for other initial conditions. One uses R (Eq 18) and notes *S* ≤ *S*_0_.

Corresponding results can be obtained if person-to-person transmission is included in the model; the proof is given in S1 Appendix. A corresponding result when birth–death dynamics are included in the model—which allows an endemic equilibrium to exist and reduces the set of disease free equilibria to a point—is an extension of a result previously shown by Wang and Liao [28] and can also be proved using Theorem 2.2 of Shuai and van den Driessche [66].

**Corollary 1.** *If human birth–death dynamics and person-to-person transmission are included in* Eq (3), *then, if* *f* *is concave, we have that*

1. *if* $R_{0}<1$, *the disease-free equilibrium* (*N*, 0, 0, 0, 0) *is globally asymptotically stable*.
2. *if* $R_{0}>1$, *then the disease-free equilibrium is unstable*.
3. *if* $R_{0}>1$, *there exists a unique endemic equilibrium*.

Stability results for the endemic equilbrium are likely possible in this scenario as well. If we do not constrain to concave dose–response functions, it can also be shown that the choice of dose–response function can affect the kinds of dynamics that can arise in an infectious disease system, such as multiple equilibria and or periodic orbits [68].

Our results strongly encourage the use of concave, low-dose linear functions. Low-dose linear functions give biologically reasonable (i.e. finite, non-zero) values for the basic reproduction number, and concavity ensures that the epidemic dynamics actually correspond to the value of $R_{0}$ for all initial conditions. Except in cases where pathogen cooperation or thresholds are specifically being considered, the choice of a concave, low-dose linear dose–response function is biologically sensible and ensures that the basic reproductive number is a useful and relevant measure of the global dynamics of the system. Choosing one of the single-hit models in Table 1 satisfies these properties.

### Leveraging environmental monitoring: Implications for infectivity inference

In addition to the uncertainty introduced by using medium- and high-dose data to estimate low-dose infectivity, as discussed in a previous section, other uncertainties associated with environmental measures should be considered. In particular, the volume of the environment (implicitly appearing in the shedding rate *α*) is difficult to measure, and the shedding rate can be highly variable depending on pathogen strain or host immune status. One solution is to identify combinations of parameters that control the dynamics, such as $R_{0}$, that we can estimate with confidence from outbreak data.

In this section, we show that when we are interested in describing the time series of prevalence, it is not necessary to know *f* or even uniquely identify the low-dose per-pathogen infectivity *π*, as long as the average dose remains within the linear regime of the dose–response function. We demonstrate this through identifiability analysis of the linear model (Eq (4)); identifiability analysis is the assessment of which parameters, or parameter combinations, can be uniquely determined from the data. The following theorem states that, given time-series prevalence data, we can identity values of parameter combinations that uniquely describe the model fit to the data. We do not need information on the specific parameter values that constitute the combinations. This result follows the work of Eisenberg et al. [16] on the SIWR model, which developed the first identifiability results for an environmentally mediated transmission model and emphasized the role of environmental monitoring in inference of the shedding rate. (In the notation of Eisenberg et al. [16], *β*_*I*_ = 0, *β*_*W*_ = *κρπ*, *k* = 1).

**Theorem 2.** *The identifiable combinations of the model given in* Eq (4) *given time series data of prevalence of infected individuals I are* *απκρ*, *ξ*, *γ* + *σ*, *and* *γσ*. *If the time series of the environmental compartment W is also observed, then* *α* *is separately identifiable*.

**Remark.** Parameters γ and σ are locally identifiable because there are only two solutions given γ + σ and γσ. In most cases, external information will resolve any ambiguity.

The proof is left to S1 Appendix. This result means that, if we have prevalence data but no environmental monitoring, the individual values of the shedding rate *α* and the pathogen infectivity *π* can vary without changing the outbreak dynamics as long as the product *απκρ* does not change. For example, because the product *απκρ* appears in the numerator of $R_{0}$, the model can be parameterized to match $R_{0}$ by balancing the low-dose slope of the dose–response function fit to data (i.e. *π*) with the shedding rate *α*. (Because *κ* and *ρ* implicitly appear in another identifiable combination, *ξ* = *μ* + *κ*(*ρ*/*V*)*N*, they cannot be adjusted without changing this combination if *μ* is well known from experimental data, although if *κ*(*ρ*/*V*)*N* ≪ *μ*, the difference may smaller than the measurement error in the data, meaning that the change is not practically identifiable). This allows the modeler to mitigate misspecification of the dose–response model. With environmental monitoring, *α* can be estimated from the data, which allows us in turn to gain more information about *π*.

**Fig 7:**
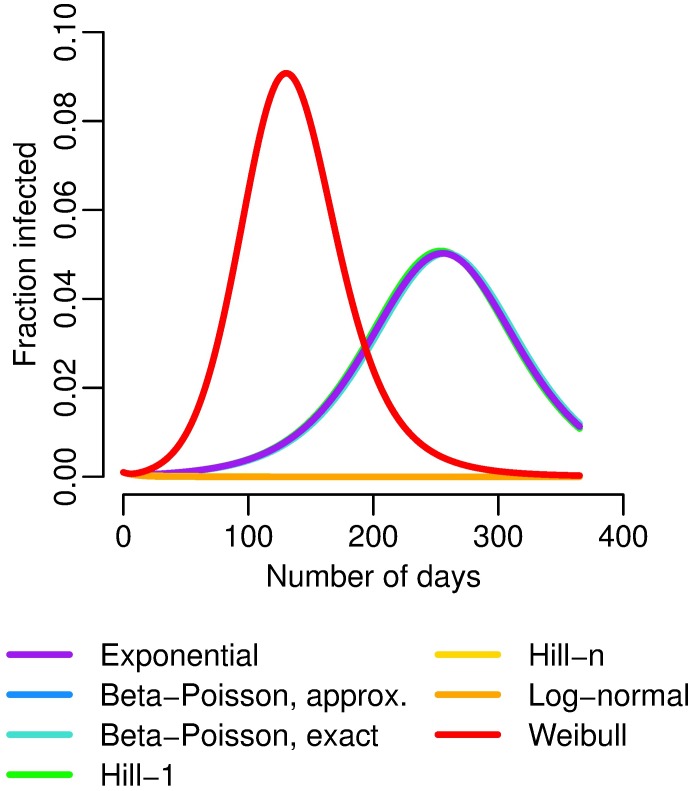
*Cryptosporidium* dose–response and dynamics with the fixed stochastic $R_{0}$. Modeled number of infected people under different dose–response relationships, where *α* is adjusted so that $R_{0}^{*}=2$ for each simulation. The exponential, exact and approximate beta–Poisson, and Hill-1 functions lie nearly on top of each other, and the Hill-*n* and lognormal functions have no outbreak.

We illustrate the identifiability results with three examples. First, we expand upon the *Cryptosporidium* example above by maintaining all of the same parameters given for Fig 2 except for the shedding rate *α*, which is set so that each simulation has $R_{0}^{*}=2$. (We use the stochastic basic reproduction number instead of the deterministic one to facilitate comparison with the models that have necessarily zero or infinite deterministic $R_{0}$.) The four low-dose linear functions (exponential, exact and approximate Beta–Poisson, and Hill-1) now give identical dynamics (Fig 7). The differences in outbreak dynamics given the differences in the fit of the dose–response functions disappear if we privilege our information about $R_{0}$ over the shedding estimates. Because of their curvature, the Hill-*n* (no outbreak), log-normal (no outbreak), and Weibull (outbreak too large) still behave badly, further highlighting the importance of non-zero, finite low-dose linearity.

**Fig 8:**
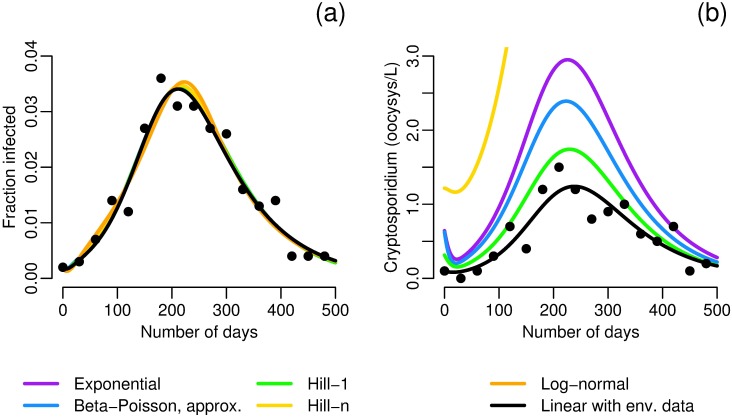
Simulated data and model fits for an outbreak of *Cryptosporidium*. In both plots the colored lines are model fits for environmentally mediated infectious disease transmission models with dose–response relationships (Eq (3)), fit only to case data, that use the indicated dose–response function. The black line is the environmentally mediated infectious disease transmission model with linear infectivity (Eq (4)), fit to both case and environmental data. a) Case data and model fits. b) Environmental data and model fits (assuming *κ* = 8, and *ρ* = 0.15 [47] in order to estimate *W* in those models that do not observe the environmental data). The environmental estimate of the log-normal model, with an initial condition of 8.4 oocysts/L, is outside of the scale of the plot. Additional simulation details are given in S1 Appendix.

In the second example, we use simulated data of an extended, low-prevalence outbreak of *Cryptosporidium* in a village of 1,000 situated by a small body of water. Details for the simulation of data are given in S1 Appendix. The number of cases of cryptosporidosis is recorded monthly. The model (Eq (3)) is fit to the data using each of functional forms found in Fig 2a except for the Weibull, which did not converge, and the exact beta–Poisson. Parameter combinations *ακρ*, *ξ*, *γ*, and *σ* are estimated. All of the models are able to fit the data reasonably well (Fig 8a). However, the models give very different estimates of concentration of pathogens in the water (Fig 8b) under basic assumptions of the frequency and volume of water consumption and an initially fully susceptible population. If we additionally monitor the environment, then the shedding rate *α* is separately identifiable, and we can see from the lack of fit in Fig 8b that the models with dose–response functions do not capture this parameter or the environmental data correctly. The differences in low-dose infectivity among the dose–response forms can be offset by other parameters when the model is fit the case data alone, but this is not possible when the environment is also measured and daily volume of water ingested is reasonably well known. In both Fig 8a and 8b, we plot the best-fit (fit to both data sets) linear model (Eq (4)), showing that the model with linear infectivity suffices to capture the dynamics. This example demonstrates two important points. First, the additional information available in environmental monitoring can be a powerful tool for parameter estimation, and, second, fixing a dose–response function is essentially a constraint on the identifiable parameter combinations that may lead to spurious estimation of other parameters.

#### Case study: The Milwaukee cryptosporidosis outbreak

Finally, we apply our methods to analyze the cryptosporidosis outbreak in Milwaukee, Wisconsin in March and April of 1993 [69]. The outbreak was attributed to failure of one of two water treatment plants for the city. The failure occurred on approximately March 23, and the treatment was shut down on April 9th. The outbreak began shortly after the treatment plant failure and cases of watery diarrhea returned to pre-outbreak levels by April 20th. After the outbreak, researchers surveyed households and recorded remembered onset, duration, and character of diarrhea. Additionally, concentration of *Cryptosporidium* oocysts was measured in two water samples taken on March 25th and April 9th, and turbidity was measured daily.

**Fig 9:**
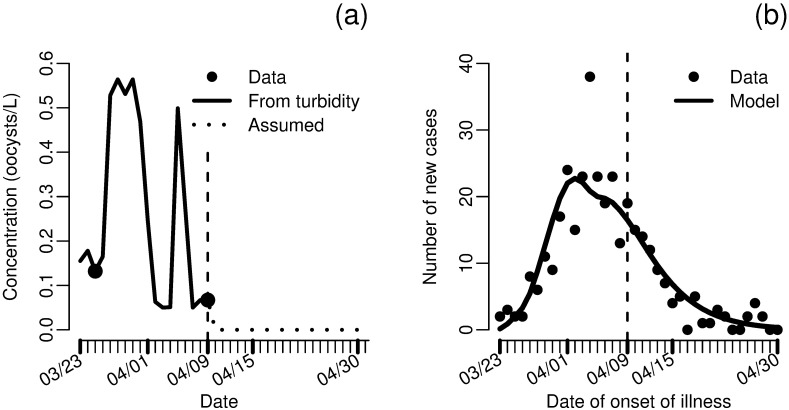
Data and model fits for the 1993 Milwaukee cryptosporidosis outbreak. a) Concentration of *Cryprosporidium* in treated water from data and estimated from turbidity. b) Fit of the environmentally mediated infectious disease model (forced by estimated *Cryptosporidium* concentration and incorporating two exposed compartments) to incidence of new cases of diarrhea. Parameters *πκρ* = 0.0507 and *σ* = 0.134 were estimated in the model fit. Additional simulation details are given in S1 Appendix.

Previous studies have used a variety of models for parameter estimation for this outbreak (e.g. [13, 70]), but we take a slightly different approach. We assume a linear relationship between turbidity and *Cryptosporidium* concentration. Although the correlation between turbidity and pathogen concentrations is poor in general [71], turbidity is a much better predictor of specific pathogen contamination when that contamination is due to an event that also increases turbidity. This estimated concentration (Fig 9a) was used as the environmental compartment in a model analogous to Eq (4) but with two exposed compartments, i.e. the environmental data was used as a forcing function input rather than data to be modeled (exact model equations given in S1 Appendix). This forced model was fit to incidence of new cases (Fig 9b). The model captures the data well, both in the initial phase and after the treatment plant was closed, although the peak does not reach quite as high as suggested by the data. As the data were self-reported and subject to recall bias, this discrepancy is tolerable. Error may also be introduced by neglecting person-to-person transmission, although other analyses have suggested that secondary transmission was responsible for at most 5–10% of cases [13, 72].

This forcing function approach may be valuable in the future when there is environmental exposure data but a great deal of uncertainty in the environmental pathway between shedding and ingestion. Here, the variation in the turbidity is likely caused by heterogeneity in the environment or attempts to fix the treatment plant, neither of which would be the captured by the original model. However, because we are estimating exposures from the environmental data, it is not necessary to consider shedding or the fate of the pathogens in the environment.

We estimate the combination *κρπ* to be approximately 0.05. If we approximate *κρ*, the daily volume of water ingested per person, to be 1.2 L [47], then we estimate *π* to be approximately 0.04, far larger than the *π* estimates from Fig 2a, which ranged from 4E-3 to 7E-3 (see Table 3 for exact values). This estimate suggests that the strain of *Cryptosporidium* seen in the Milwaukee outbreak (genotype Ib [73, 74]) was an order of magnitude more infectious than that studied by DuPont et al. [45], more similar to the TAMU strain than the Iowa strain [75]. There are a couple sources of uncertainty here, including possible differences in susceptibility between the study population of DuPont et al. [45] and the residents of Milwaukee. Another source of uncertainty is potential deviation from the assumption of linearity between turbidity and pathogen concentration. In this case, it is more reasonable to expect a sublinear rather than superlinear relationship between pathogen concentration and turbidity; a sublinear relationship would suggest a more, rather than less, infective strain, and, therefore, our estimate is conservative. By using the identifiable information in the data rather than taking a dose–response relationship as a given, we were able to make inference about the pathogen infectivity. In future outbreaks, our methods may be able to provide information that could lead to enhanced mitigation strategies.

### Conclusion

When using environmentally mediated infectious disease transmission models, a linear infectivity parameter in lieu of a dose–response function is sufficient when transmission dynamics occur in a low-average-dose setting. In the case that a dose–response function is in fact needed, we should only consider low-dose linear, concave, single-hit (i.e. biologically plausible) functions (e.g. the exponential, beta–Poisson, or, with caveats, Hill-1) and be cognizant of the fact that medium- and high-dose exposures are used to fit the dose–response models that are used to examine the impact of low-dose environmental exposures. We should not automatically accede to the best-fit model presented in the literature, especially when multiple functions fit well, but rather acknowledge the uncertainty across functions in the low-dose regime and conduct sensitivity analyses. Using a dose–response function also requires us to consider a biological basis for separating the rate of contact with the environment from the per-exposure pick-up rate.

The uncertainty in the low-dose infectivity parameter and in aspects of the environment itself can be better managed by considering the identifiable combinations of the model. Because the shedding rate and the infectivity, along with the contact rate and pick-up volume, occur in an identifiable product, their individual values do not affect the model dynamics, as long as the value of the parameter combination is preserved. This product can be estimated from case data or possibly from the basic reproductive number $R_{0}$. The *a priori* choice of a dose–response function amounts to a constraint on the value of the infectivity, which, if not appropriate for the particular outbreak, will lead to spurious estimates of other parameters. Alternatively, environmental monitoring provides additional information that can be used to identify shedding rates and, via this identifiable product, pathogen infectivity.

## Supporting information

S1 Appendix Includes alternate models, additional dose–response model fits for influenza, rotavirus, and *Salmonella typhi*, parameter estimates for all dose–response models, additional details for the stochastic basic reproduction number, global dynamics results when including person-to-person transmission, and the proof of the identifiability theorem. (PDF)

## References

[1] Codeço CT. Endemic and epidemic dynamics of cholera: the role of the aquatic reservoir. BMC Infectious Diseases. 2001;1:1 10.1186/1471-2334-1-1 11208258 PMC29087

[2] Capasso V, Paveri-Fontana SL. A mathematical model for the 1973 cholera epidemic in the European Mediterranean region. Revue d’épidémiologie et de santé publique. 1979;27(2):121–32. 538301

[3] Hartley DM, Morris JG, Smith DL. Hyperinfectivity: A critical element in the ability of V. cholerae to cause epidemics? PLOS Medicine. 2006;3(1):63–69. 10.1371/journal.pmed.0030007 PMC1298942 16318414

[4] Bertuzzo E, Azaele S, Maritan A, Gatto M, Rodriguez-Iturbe I, Rinaldo A. On the space-time evolution of a cholera epidemic. Water Resources Research. 2008;44(1):1–8. 10.1029/2007WR006211

[5] Bertuzzo E, Casagrandi R, Gatto M, Rodriguez-Iturbe I, Rinaldo A. On spatially explicit models of cholera epidemics. Journal of the Royal Society Interface. 2010;7(43):321–333. 10.1098/rsif.2009.0204 PMC2842613 19605400

[6] Miller Neilan RL, Schaefer E, Gaff H, Fister KR, Lenhart S. Modeling Optimal Intervention Strategies for Cholera. Bulletin of Mathematical Biology. 2010;72(8):2004–2018. 10.1007/s11538-010-9521-8 20204710

[7] Andrews JR, Basu S. Transmission dynamics and control of cholera in Haiti: An epidemic model. The Lancet. 2011;377(9773):1248–1255. 10.1016/S0140-6736(11)60273-0 PMC3172163 21414658

[8] Righetto L, Bertuzzo E, Casagrandi R, Gatto M, Rodriguez-Iturbe I, Rinaldo A. Modelling human movement in cholera spreading along fluvial systems. Ecohydrology. 2011;4:49–55. 10.1002/eco.122

[9] Mari L, Bertuzzo E, Righetto L, Casagrandi R, Gatto M, Rodriguez-Iturbe I, et al Modelling cholera epidemics: The role of waterways, human mobility and sanitation. Journal of the Royal Society, Interface. 2012;9(67):376–388. 10.1098/rsif.2011.0304 21752809 PMC3243392

[10] Eisenberg JN, Seto EY, Colford JM, Olivieri A, Spear RC. An analysis of the Milwaukee cryptosporidiosis outbreak based on a dynamic model of the infection process. Epidemiology. 1998;9(3):255–263. 10.1097/00001648-199805000-00008 9583416

[11] Brookhart MA, Hubbard AE, Van Der Laan MJ, Colford JM, Eisenberg JNS. Statistical estimation of parameters in a disease transmission model: Analysis of a Cryptosporidium outbreak. Statistics in Medicine. 2002;21(23):3627–3638. 10.1002/sim.1258 12436460

[12] Eisenberg JNS, Brookhart MA, Rice G, Brown M, Colford JM. Disease transmission models for public health decision making: Analysis of epidemic and endemic conditions caused by waterborne pathogens. Environmental Health Perspectives. 2002;110(8):783–790. 10.1289/ehp.02110783 12153759 PMC1240949

[13] Eisenberg JNS, Lei X, Hubbard AH, Brookhart MA, Colford JM. The role of disease transmission and conferred immunity in outbreaks: Analysis of the 1993 Cryptosporidium outbreak in Milwaukee, Wisconsin. American Journal of Epidemiology. 2005;161(1):62–72. 10.1093/aje/kwi005 15615916

[14] Li S, Spicknall IH, Koopman JS, Eisenberg JNS. Dynamics and control of infections transmitted from person to person through the environment. American Journal of Epidemiology. 2009;170(2):257–265. 10.1093/aje/kwp116 19474071

[15] Tien JH, Earn DJD. Multiple transmission pathways and disease dynamics in a waterborne pathogen model. Bulletin of Mathematical Biology. 2010;72(6):1506–33. 10.1007/s11538-010-9507-6 20143271

[16] Eisenberg MC, Robertson SL, Tien JH. Identifiability and estimation of multiple transmission pathways in cholera and waterborne disease. Journal of Theoretical Biology. 2013;324:84–102. 10.1016/j.jtbi.2012.12.021 23333764

[17] Eisenberg MC, Shuai Z, Tien JH, van den Driessche P. A cholera model in a patchy environment with water and human movement. Mathematical Biosciences. 2013;246(1):105–112. 10.1016/j.mbs.2013.08.003 23958383

[18] Robertson SL, Eisenberg MC, Tien JH. Heterogeneity in multiple transmission pathways: modelling the spread of cholera and other waterborne disease in networks with a common water source. Journal of Biological Dynamics. 2013;7(1):254–75. 10.1080/17513758.2013.853844 24303905

[19] Tien JH, Shuai Z, Eisenberg MC, van den Driessche P. Disease invasion on community networks with environmental pathogen movement. Journal of Mathematical Biology. 2014;70(5):1065–1092. 10.1007/s00285-014-0791-x 24792228

[20] Brouwer AF, Eisenberg MC, Remais JV, Collender PA, Meza R, Eisenberg JNS. Modeling biphasic environmental decay of pathogens and implications for risk analysis. Environmental Science & Technology. 2016;51(4):2186–2196. 10.1021/acs.est.6b04030 PMC5789392 28112914

[21] Haas CN. Estimation of risk due to low doses of microorganisms: a comparison of alternative methodologies. American Journal of Epidemiology. 1983;118(4):573–582. 10.1093/oxfordjournals.aje.a113662 6637984

[22] Haas CN, Rose JB, Gerba C, Regli S. Risk assessment of virus in drinking water. Risk Analysis. 1993;13(5):545–52. 10.1111/j.1539-6924.1993.tb00013.x 8259444

[23] Teunis PFM, van der Heijden OG, van der Giessen JWB, Havelaar AH. The dose-response relation in human volunteers for gastro-intestinal pathogens. Bilthoven, The Netherlands: National Institute of Public Health and the Environment; 1996.

[24] Gaddum JH. Reports on Biological Standards. III. Methods of Biological Assay Depending on a Quantal Response. London: H.M. Stationary Office; 1933.

[25] Coleman M, Marks H. Topics in dose-response modeling. Journal of Food Protection. 1998;61(11):1550–9. 10.4315/0362-028X-61.11.1550 9829203

[26] Holcomb DL, Smith MA, Ware GO, Hung YC, Brackett RE, Doyle MP. Comparison of six dose-response models for use with food-borne pathogens. Risk Analysis. 1999;19(6):1091–100. 10.1111/j.1539-6924.1999.tb01130.x 10765449

[27] Food and Agriculture Organization of the United Nations, World Health Organization. Hazard characterization for pathogens in food and water: Guidelines Geneva: World Health Organizations; 2003.

[28] Wang J, Liao S. A generalized cholera model and epidemic–endemic analysis. Journal of Biological Dynamics. 2012;6(2):568–589. 10.1080/17513758.2012.658089 22873606

[29] Haas CN, Rose JB, Gerba CP. Quantitative Microbial Risk Assessment. Hoboken, NJ: John Wiley & Sons, Inc; 2014.

[30] Nilsen V, Wyller J. QMRA for Drinking Water: 1. Revisiting the Mathematical Structure of Single-Hit Dose-Response Models. Risk Analysis. 2016;36(1):145–162. 10.1111/risa.12389 26812257

[31] Crump KS, Hoel DG, Langley CH, Peto R. Fundamental Carcinogenic Processes and Their Implications for Low Dose Risk Assessment. Cancer Research. 1976;36:2973–2979. 975067

[32] Crawford M, Wilson R. Low-dose linearity: The rule or the exception? Human and Ecological Risk Assessment. 1996;2(2):305–330. 10.1080/10807039609383610

[33] Rubin L. Bacterial Colonization and Infection Resulting from Multiplication of a Single Organism Clinical Infectious Diseases. 1987;9(3):488–493. 10.1093/clinids/9.3.488 3299635

[34] Nilsen V, Wyller J. QMRA for Drinking Water: 2. The Effect of Pathogen Clustering in Single-Hit Dose-Response Models. Risk Analysis. 2016;36(1):163–181. 26812258 10.1111/risa.12528

[35] Teunis PFM, Moe CL, Liu P, Miller SE, Lindesmith L, Baric RS, et al Norwalk virus: How infectious is it? Journal of Medical Virology. 2008;80(8):1468–1476. 10.1002/jmv.21237 18551613

[36] Schmidt PJ. Norovirus Dose-Response: Are Currently Available Data Informative Enough to Determine How Susceptible Humans Are to Infection from a Single Virus? Risk Analysis. 2015;35(7):1364–1383. 10.1111/risa.12323 25522208

[37] Teunis PFM, Havelaar AH. The Beta Poisson dose-response model is not a single-hit model. Risk Analysis. 2000;20(4):513–520. 10.1111/0272-4332.204048 11051074

[38] Furumoto WA, Mickey R. A mathematical model for the infectivity-dilution curve of tobacco mosaic virus: Experimental tests. Virology. 1967;32(2):224–233. 10.1016/0042-6822(67)90271-1 6025876

[39] Furumoto WA, Mickey R. A mathematical model for the infectivity-dilution curve of tobacco mosaic virus: Theoretical considerations. Virology. 1967;32(2):216–223. 10.1016/0042-6822(67)90271-1 6025875

[40] Schmidt PJ, Pintar KDM, Fazil AM, Topp E. Harnessing the theoretical foundations of the exponential and beta-poisson dose-response models to quantify parameter uncertainty using markov chain monte carlo. Risk Analysis. 2013;33(9):1677–1693. 10.1111/risa.12006 23311599

[41] Diekmann O, Heesterbeek JAP, Metz JAJ. On the definition and the computation of the basic reproduction ratio R0 in models for infectious diseases in heterogeneous populations. Journal of Mathematical Biology. 1990;28:365–382. 10.1007/BF00178324 2117040

[42] van den Driessche P, Watmough J. Reproduction numbers and sub-threshold endemic equilibria for compartmental models of disease transmission. Mathematical Biosciences. 2002;180:29–48. 10.1016/S0025-5564(02)00108-6 12387915

[43] Raue A, Kreutz C, Maiwald T, Bachmann J, Schilling M, Klingmüller U, et al Structural and practical identifiability analysis of partially observed dynamical models by exploiting the profile likelihood. Bioinformatics. 2009;25(15):1923–1929. 10.1093/bioinformatics/btp358 19505944

[44] Luebeck G, Meza R. Bhat: General likelihood exploration; 2013. R package version 0.9-10. Available from: http://CRAN.R-project.org/package=Bhat.

[45] DuPont HL, Chappell CL, Sterling CR, Okhuysen PC, Rose JB, Jakubowski W. The Infectivity of Cryptosporidium parvum in Healthy Volunteers. New England Journal of Medicine. 1995;332(13):855–859. 10.1056/NEJM199503303321304 7870140

[46] Centers for Disease Control and Prevention. Parasites—Cryptosporidium—Illness & Symptoms; 2016. http://www.cdc.gov/parasites/crypto/illness.html. Accessed June 24, 2016.

[47] U S Environmental Protection Agency. Exposure Factors Handbook: 2011 Edition. Washington DC: National Center for Environmental Assessment; 2011.

[48] Chappell CL, Okhuysen PC, Sterling CR, DuPont HL. Cryptosporidium parvum: intensity of infection and oocyst excretion patterns in healthy volunteers. The Journal of Infectious Diseases. 1996;173(1):232–236. 10.1093/infdis/173.1.232 8537664

[49] Peng X, Murphy T, Holden NM. Evaluation of the effect of temperature on the die-off rate for Cryptosporidium parvum oocysts in water, soils, and feces. Applied and Environmental Microbiology. 2008;74(23):7101–7107. 10.1128/AEM.01442-08 18849452 PMC2592903

[50] Hornick RB, Music SI, Wenzel R, Cash R, Libonati JP, Snyder MJ, et al The Broad Street pump revisited: response of volunteers to ingested cholera vibrios. Bulletin of the New York Academy of Medicine. 1971;47(10):1181–91. 5286453 PMC1749960

[51] Centers for Disease Control and Prevention. Cholera—Vibrio cholerae infection; 2016. http://www.cdc.gov/cholera/general/index.html. Accessed June 24, 2016.

[52] DuPont HL, Hornick RB, Dawkins AT, Snyder MJ, Formal SB. The response of man to virulent Shigella flexneri 2a. The Journal of Infectious Diseases. 1969;119(3):296–299. 10.1093/infdis/119.3.296 5780532

[53] DuPont HL, Hornick RB, Snyder MJ, Libonati JP, Samuel BF, Gangarosa EJ. Immunity in shigellosis. II. Protection induced by oral live vaccine or primary infection. The Journal of Infectious Diseases. 1972;125(1):12–16. 10.1093/infdis/125.1.12 4550416

[54] Centers for Disease Control and Prevention. Shigella–Shigellosis; 2016. http://www.cdc.gov/shigella/general-information.html. Accessed June 24, 2016.

[55] El-Sharkawi F, El-Attar L, Gawad AA, Molazem S. Some environmental factors affecting survival of fecal pathogens and indicator organisms in seawater. Water Science and Technology. 1989;21(1):115–120.

[56] Ghosh AR, Sehgal SC. Survivability of Shigella dysenteriae type 1 & S. flexneri 2a in laboratory conditions simulating aquatic environment. Indian Journal of Medical Research. 2001;114:199–206. 12040763

[57] Weir M. Dose-Response Modeling and Use: Challenges and Uncertainties in Environmental Exposure In: Pillai SD, Nakatsu CH, Miller RV, Yates MV, editors. Manual of Environmental Microbiology. 4th ed Washington DC: American Society of Microbiology; 2015 p. 3.5.3–1–3.5.3–17.

[58] Dietz K, Schenzle D. Mathematical Models for Infectious Disease Statistics In: Atkinson AC, Fienberg SE, editors. Mathematical Models for Infectious Disease Statistics. New York, NY: Springer New York; 1985 p. 167–204.

[59] Pujol JM, Eisenberg JE, Haas CN, Koopman JS. The effect of ongoing exposure dynamics in dose response relationships. PLOS Computational Biology. 2009;5(6):1–12. 10.1371/journal.pcbi.1000399 PMC2685010 19503605

[60] Mayer BT, Koopman JS, Ionides EL, Pujol JM, Eisenberg JNS. A dynamic dose-response model to account for exposure patterns in risk assessment: a case study in inhalation anthrax. Journal of The Royal Society Interface. 2011;8(57):506–517. 10.1098/rsif.2010.0491 PMC3061128 21068030

[61] Brachman PS, Kaufman AF, Dalldorf FG. Industrial inhalation Anthrax. Bacteriological reviews. 1966;30(3):646–659 4958345 10.1128/br.30.3.646-659.1966 PMC378258

[62] Bani-Yaghoub M, Gautam R, Shuai Z, van den Driessche P, Ivanek R. Reproduction numbers for infections with free-living pathogens growing in the environment. Journal of Biological Dynamics. 2012;6(2):923–940. 10.1080/17513758.2012.693206 22881277

[63] Breban R. Role of environmental persistence in pathogen transmission: a mathematical modeling approach. Journal of Mathematical Biology. 2013;66(3):535–546. 10.1007/s00285-012-0520-2 22382994 PMC7079992

[64] Allen LJS, Lahodny GE. Extinction thresholds in deterministic and stochastic epidemic models. Journal of Biological Dynamics. 2012;6(2):590–611. 10.1080/17513758.2012.665502 22873607

[65] Laukó IG. Stability of disease free sets in epidemic models. Mathematical and Computer Modelling. 2006;43(11–12):1357–1366.

[66] Shuai Z, van den Driessche P. Global stability of infectious disease models using Lyapunov funtions. SIAM Journal on Applied Mathematics. 2013;73(4):1513–1532. 10.1137/120876642

[67] Lasalle JP. Some Extensions of Liapunov’s Second Method. IRE Transactions on Circuit Theory. 1960;7(4):520–527. 10.1109/TCT.1960.1086720

[68] Dunworth JB. Nonlinear Incidence of Waterborne Diseases. The Ohio State University; 2011.

[69] Mac Kenzie WR, Hoxie NJ, Proctor ME, Gradus MS, Blair KA, Peterson DE, et al A Massive Outbreak in Milwaukee of Cryptosporidium Infection Transmitted through the Public Water Supply. New England Journal of Medicine. 1994;331(3):161–167. 10.1056/NEJM199407213310304 7818640

[70] Gupta M, Haas CN. The Milwaukee Cryptosporidium outbreak: Assessment of incubation time and daily attack rate. Journal of Water and Health. 2004;2(2):59–69. 15387130

[71] Ministerial Technical Advisory Committee. Turbidity and Microbial Risk in Drinking Water Minister of Health of the Province of British Columbia. 2008.

[72] MacKenzie WR, Schell WL, Blair KA, Addiss DG, Peterson DE, Hoxie NJ, Kazmierczak JJ, Davis JP. Massive outbreak of waterborne cryptosporidium infection in Milwaukee, Wisconsin: recurrence of illness and risk of secondary transmission. Clinical Infectious Diseases. 1995;21(1):57–62. 10.1093/clinids/21.1.57 7578760

[73] Sulaiman IM, Lal AA, Xiao L. A Population Genetic Study of the Cryptosporidium parvum Human Genotype Parasites. Journal of Eukaryotic Microbiology. 2001;48(s1):24s–27s. 10.1111/j.1550-7408.2001.tb00441.x 11906066

[74] Zhou L, Singh A, Jiang J, Xiao L. Molecular Surveillance of Cryptosporidium spp. in Raw Wastewater in Milwaukee: Implications for Understanding Outbreak Occurrence and Transmission Dynamics. Journal of Clinical Microbiology. 2003;41(11):5254–5257. 10.1128/JCM.41.11.5254-5257.2003 14605176 PMC262506

[75] Messner MJ, Chappell CL, Okhuysen PC. Risk assessment for Cryptosporidium: a hierarchical Bayesian analysis of human dose response data. Water Research. 2001;35(16):3934–3940. 10.1016/S0043-1354(01)00119-1 12230176

