# Correction: Dose-response relationships for environmentally mediated infectious disease transmission models

**DOI:** 10.1371/journal.pcbi.1005765

**Published:** 2017-09-20

**Authors:** The PLOS Computational Biology Staff

The funding statement for this article should read as follows:

“This work was funded under the Models of Infectious Disease Agent Study (MIDAS) program (www.nigms.nih.gov/Research/SpecificAreas/MIDAS/Pages/default.aspx) within the National Institute of General Medical Sciences of the National Institutes of Health (grant number U01GM110712) and under the National Science Foundation Water Sustainability and Climate Program (grant 1360330). AFB, MCE, RM, JNSE received funding from one or both of these sources. The funders had no role in study design, data collection and analysis, decision to publish, or preparation of the manuscript.”
