# Supplementary material for: Dose-response relationships for environmentally mediated infectious disease transmission models
Source: PLoS Comput Biol. 2017 Apr 7;13(4):e1005481. doi: 10.1371/journal.pcbi.1005481 (PMC5400279; doi:10.1371/journal.pcbi.1005481)
Supplement: S1 Appendix — (PDF) [file pcbi.1005481.s001.pdf]

Dose–response Relationships for Environmentally Mediated Infectious Disease Transmission Models: Appendix

1. Alternate models

We consider several variations on the environmentally mediated infectious disease transmission model with dose–response (Eqs. (3)). In the first, we count the number W^* rather than the concentration W of pathogens. This requires a slight redefinition of the shedding α and pick-up ρ parameters, because it shifts the implicit volume of the environment from incorporation in α to ρ. We write these redefined parameters as $\alpha^* = \alpha V$ and $\rho^* = \rho/V$.

$$
\begin{aligned}
\dot{S} &= -\kappa f(\rho^* W^*) S \\
\dot{E} &= \kappa f(\rho^* W^*) S - \sigma E \\
\dot{I} &= \sigma E - \gamma I \\
\dot{R} &= \gamma I \\
\dot{W}^* &= \alpha^* I - \xi W^*
\end{aligned}
\tag{S1}
$$

Next, we include (frequency dependent) person-to-person transmission at rate β.

$$
\begin{aligned}
\dot{S} &= -(\kappa f(\rho W) + \beta I/N) S \\
\dot{E} &= (\kappa f(\rho W) + \beta I/N) S - \sigma E \\
\dot{I} &= \sigma E - \gamma I \\
\dot{R} &= \gamma I \\
\dot{W} &= \alpha I - \xi W
\end{aligned}
\tag{S2}
$$

Then, we include human birth–death, occurring at rate ν.

$$
\begin{aligned}
\dot{S} &= \nu(1 - S) - \kappa f(\rho W) S \\
\dot{E} &= \kappa f(\rho W) S - (\sigma + \nu) E \\
\dot{I} &= \sigma E - (\gamma + \nu) I \\
\dot{R} &= \gamma I - \nu R \\
\dot{W} &= \alpha I - \xi W
\end{aligned}
\tag{S3}
$$

Finally, we extend the current model to include m exposed compartments,

$$
\begin{aligned}
\dot{S} &= -\kappa f(\rho W)S \\
\dot{E}_1 &= \kappa f(\rho W)S - m\sigma E_1 \\
\dot{E}_2 &= m\sigma E_1 - m\sigma E_2 \\
&\vdots \\
\dot{E}_i &= m\sigma E_{i-1} - m\sigma E_i \\
&\vdots \\
\dot{I} &= m\sigma E_m - \gamma I \\
\dot{R} &= \gamma I \\
\dot{W} &= \alpha I - \xi W.
\end{aligned} \tag{S4}
$$

2. Example details

In the example (Fig. 8) where a transmission model with each of the dose–response functional forms were fit to simulated data, the data were simulated in the following way. The model in Eqs. (3) was run with parameters N=1000, κ=8, ρ=0.15, σ=0.2, γ=0.1, V=4E8, α=6E5/V, μ=0.04, and and initial conditions $S(0)$=997, $I(0)$=3, $E(0)$=2, $R(0)$=0, and $W(0)$=0.05. Case data were simulated from the model trajectory by drawing from a binomial distribution with size N and probability given by the fraction of the population infected in the model trajectory on that day. Environmental concentration data were similarly simulated by drawing a number of oocysts per 10L from a Poisson distribution with mean determined from the model trajectory of the environment (modeled concentration times 10L). Computation was done in R (v3.3.1), and the seeds were set to 0 and 1 for the environmental monitoring and case data, respectively. Parameter combinations $\alpha\kappa\rho$, $\xi = \mu + \kappa\rho N/V$, γ, and σ, as well as initial conditions $E(0)$ and $W(0)S(0)/\alpha$ were estimated using the maximum likelihood approach described in the main text; the initial number of observed cases was used for $I(0)$. We assumed $S(0) = N - I(0) - E(0)$ (no prior immunity) and that N was known.

The model used for the Milwaukee cryptosporidosis outbreak (Fig. 9) uses the *Cryptosporidium* and turbidity data to estimate a time course of *Cryptospordium* concentration $T(t)$ in the water supplied to homes. This model also uses two exposed compartments. Because the data is new onset of symptoms rather than infection, we only keep track of cumulative new cases $Y(t)$. We assume new cases data K_i on day t_i comes from a binomial distribution with size N and probability $(Y(t_i) - Y(t_i - 1))/N$.

$$\begin{aligned}
\dot{S} &= -\kappa\rho T S \\
\dot{E}_1 &= \kappa\rho T S - 2\sigma E_1 \\
\dot{E}_2 &= 2\sigma E_1 - 2\sigma E_2 \\
\dot{Y} &= 2\sigma E_2
\end{aligned} \tag{S5}$$

3. Dose–response model fits

Dose–response functions and corresponding dynamics for influenza, rotavirus, and *Salmonella typhi* are shown in Figs. S1, S2, and S3 respectively. Estimated parameters and negative log-likelihoods for the maximum-likelihood estimators of the seven dose–response functions and six pathogens considered are given in Table S1.

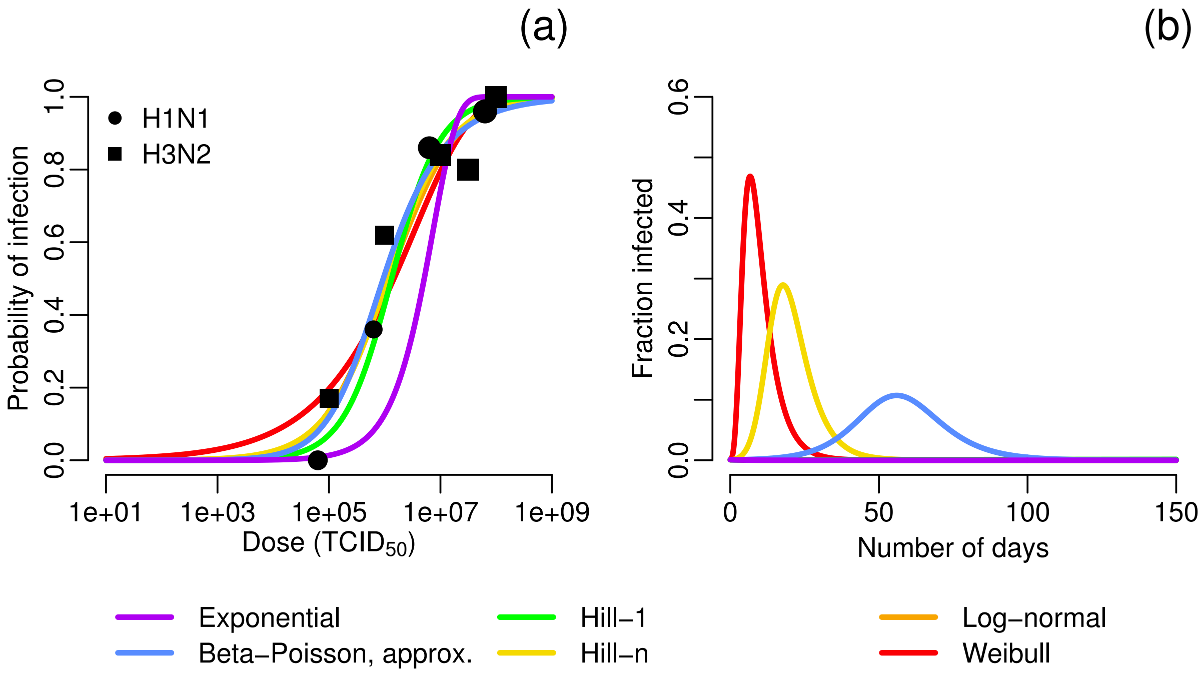

Figure S1: **Influenza dose–response and dynamics.** a) Maximum-likelihood estimates of dose–response functions for Influenza virus (H1N1 and H3N2). Data from [S1, S2]; sizes of data points are proportional to sample size. b) Modeled fraction of infected people under different influenza dose–response relationships. Model parameters are $N = 1000$, σ= 1/2, γ=1/5, κ=8, ρ= 252, $V = 6E5$, α=1E6/V, μ=8.64 [S3].

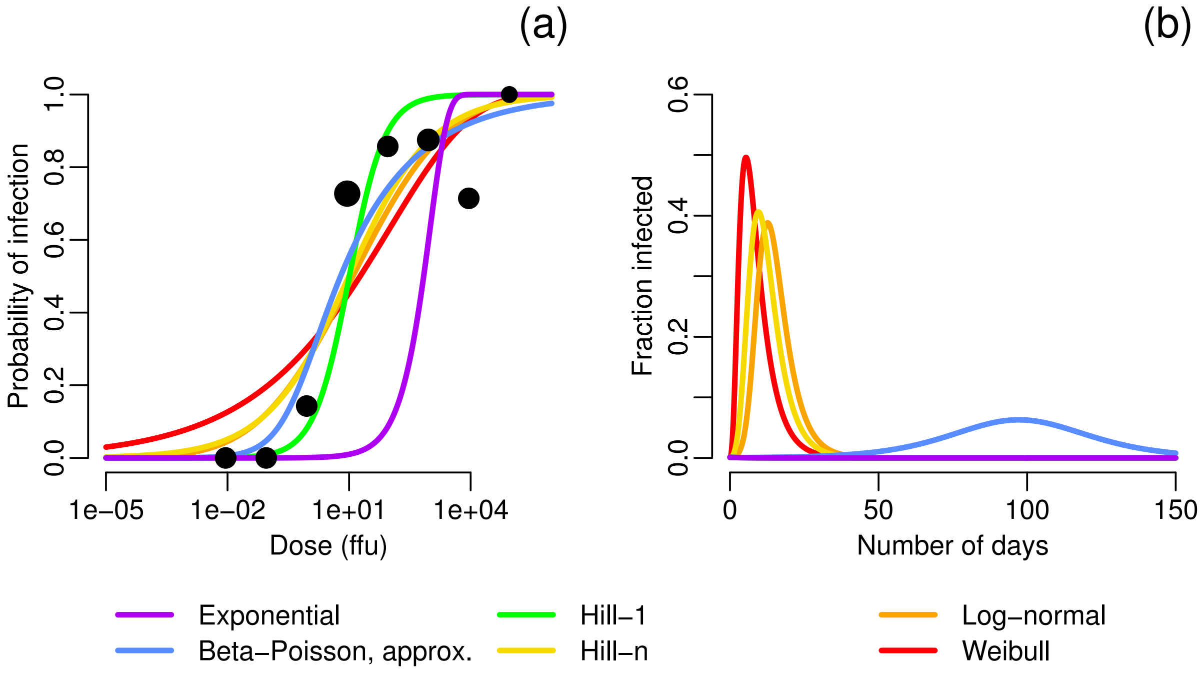

Figure S2: **Rotavirus dose–response and dynamics.** a) Maximum-likelihood estimates of dose–response functions for rotavirus. Data from [S4]; sizes of data points are proportional to sample size. b) Modeled fraction of infected people under different rotavirus dose–response relationships. Model parameters are $N = 1000$, σ= 1/2, γ=1/5, κ=8, ρ= 0.15, $V = 4E8$, α=5E4/V, μ=0.23.

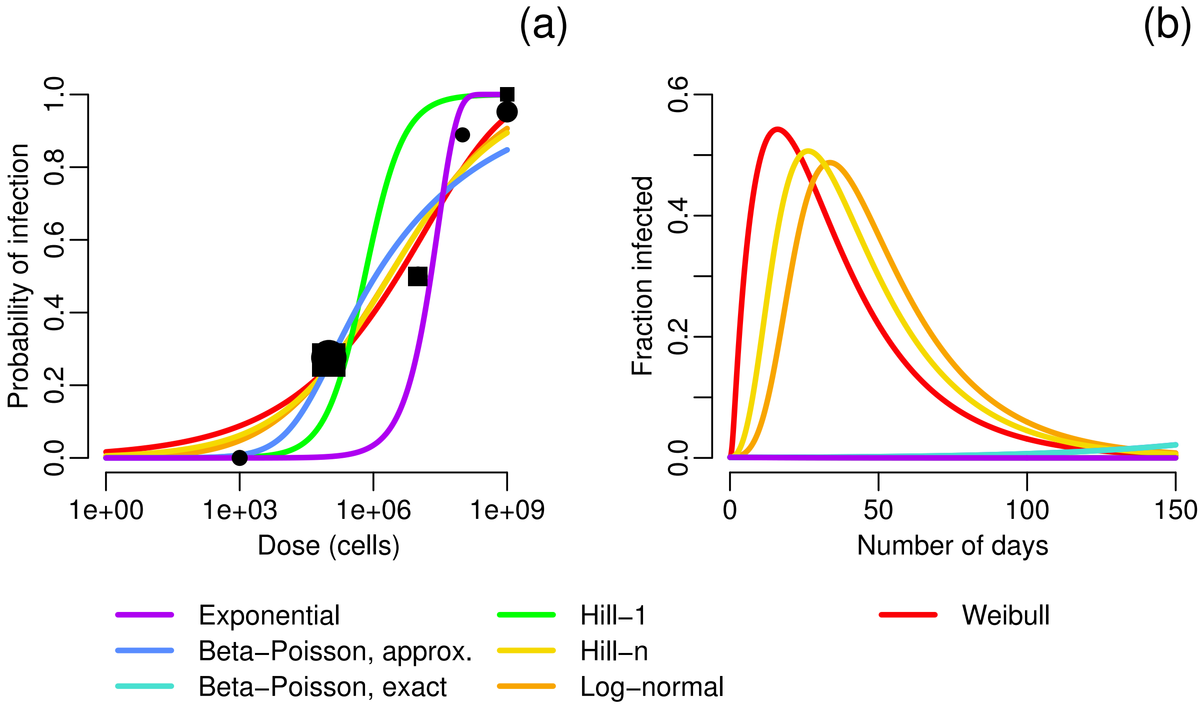

Figure S3: ***Salmonella typhi*** **dose–response and dynamics.** a) Maximum-likelihood estimates of dose–response functions for *Salmonella typhi*. Data from [S5, S6]; sizes of data points are proportional to sample size. b) Modeled fraction of infected people under different *Salmonella typhi* dose–response relationships. Model parameters are $N = 1000$, σ= 1/10, γ=1/25, κ=3, ρ= 2, $V = 2E3$, α=1E4/V, μ=1/30.

Table S1: Best fit parameters (negative log-likelihood) for the given pathogens and dose–response functions. The negative log-likelihood that admits the best fit by the Akaike information criterion is in bold. †: The exact beta–Poisson model is not fit when the units are not individual pathogens.

Pathogen	Exponential	Exact beta–Poisson	Approximate beta–Poisson	Hill-1	Hill-n	Log-normal	Weibull	Reference
Cryptosporidium parvum	$\pi = 4.005$E-3<br>(**12.5872**)	$\pi = 4.718$E-3, $\beta = 9.046$E2<br>(12.5514)	$\pi = 4.702$E-3, $\beta = 9.042$E2<br>(12.5513)	$\pi = 7.187$E-3<br>(12.7919)	$\pi = 6.985$E-3, $n = 1.229$E0<br>(12.6777)	$\mu = 4.954E1$, $\sigma = 1.367E0$<br>(12.6487)	$\pi = 4.010$E-3, $k = 8.478$E-1<br>(12.4922)	[S7]
Influenza (H3N2, H1N1)	$\pi = 1.355$E-7<br>(84.5201)	†<br>†	$\pi = 1.416$E-6, $\beta = 4.100$E6<br>(**56.8162**)	$\pi = 7.517$E-7<br>(58.9966)	$\pi = 8.916$E-7, $n = 7.733$E-1<br>(57.3795)	$\mu = 1.398$E1, $\sigma = 2.241$E0<br>(57.4477)	$\pi = 2.912$E-7, $k = 4.297$E-1<br>(59.2812)	[S1, S2]
Rotavirus	$\pi = 9.998$E-4<br>(82.126)	†<br>†	$\pi = 5.933$E-1, $\beta = 4.266$E-1<br>(**22.488**)	$\pi = 9.851$E-2<br>(32.1189)	$\pi = 1.011$E-1, $n = 4.227$E-1<br>(24.6362)	$\mu = 2.514$E0, $\sigma = 4.213$E0<br>(24.8528)	$\pi = 9.853$E-3, $k = 2.168$E-1<br>(26.7179)	[S4]
Shigella flexneri	$\pi = 6.837$E-7<br>(666.769)	$\pi = 1.141$E-2, $\beta = 1.027$E1<br>(**154.937**)	$\pi = 1.188$E-2, $\beta = 9.864$E0<br>(154.939)	$\pi = 1.333$E-4<br>(221.665)	$\pi = 2.446$E-4, $n = 1.869$E-1<br>(156.034)	$\mu = 8.324$E0, $\sigma = 8.903$E0<br>(156.123)	$\pi = 8.630$E-6, $k = 1.083$E-1<br>(156.672)	[S8, S9]
Vibrio cholerae, buffered	$\pi = 8.490$E-6<br>(63.8322)	$\pi = 1.5463$E-2, $\beta = 1.610$E1<br>(**18.2152**)	$\pi = 1.542$E-2, $\beta = 1.620$E1<br>(18.2198)	$\pi = 3.644$E-4<br>(25.6891)	$\pi = 5.641$E-3, $n = 3.207$E-1<br>(18.7615)	$\mu = 4.909$E0, $\sigma = 5.721$E0<br>(18.9054)	$\pi = 1.627$E-3, $k = 1.372$E-1<br>(19.2063)	[S10]
Salmonella typhi	$\pi = 3.567$E-8<br>(462.355)	$\pi = 8.079$E-6, $\beta = 2.169$E4<br>(189.953)	$\pi = 8.082$E-6, $\beta = 2.168$E4<br>(189.953)	$\pi = 1.559E-6$<br>(252.248)	$\pi = 4.397$E-7, $n = 3.513$E-1<br>(187.993)	$\mu = 1.462$E1, $\sigma = 4.624$E0<br>(187.393)	$\pi =$6.421E-8, $k = 2.471$E-1<br>(**186.33**)	[S5, S6]

4. Stochastic basic reproduction number

Here we prove Proposition 2. A careful accounting of the transition events and rates for the stochastic analog of the model given in Eqs. (3) is given in Table S2. Because pick-up and die-off are separate events, we do not use the parameterization $\xi = \kappa\rho N + \mu$ here. Further, it is more intuitive to use number of pathogens W^* in this derivation, although the concentration formulation is equivalent for a fixed environmental size V. Here, α and ρ are scaled as described in Section S1.

Proposition 2. *The basic reproduction number for the stochastic analog of the model given in Eqs (3) is*

$$\mathcal{R}_0^* = \frac{\alpha}{\xi} \cdot \frac{\kappa\rho N}{\kappa\rho N + \mu} \cdot f(1). \tag{S6}$$

Proof. Although there are three infected compartments (E, I, and W^*), because all exposed people necessarily become infectious, it is sufficient to consider I to be the "offspring" of W^* without explicitly considering the intermediate E.

In the notation of [S11], we write the offspring probability generating function for I given $I(0) = 1$ and $W^*(0) = 0$:

$$f_1(u_1, u_2) = \frac{\alpha u_1 u_2 + \gamma}{\alpha + \gamma} \tag{S7}$$

Similarly, we write the offspring probability generating function for W^* given $I(0) = 0$ and $W(0) = 1$:

$$f_2(u_1, u_2) = \frac{\kappa\rho N f(1) u_1 + \kappa\rho N(1 - f(1)) + \mu}{\kappa\rho N + \mu}. \tag{S8}$$

Then, the expectation matrix is

$$\mathbb{M} = \begin{bmatrix} \frac{\alpha}{\alpha+\gamma} & \frac{\kappa\rho N f(1)}{\kappa\rho N+\mu} \\ \frac{\alpha}{\alpha+\gamma} & 0 \end{bmatrix}$$

Since f_1 and f_2 are not simple functions and $\mathbb{M}$ is irreducible, then spectral radius of M determines whether the probability of ultimate extinction is 1 or less than 1. We have

$$\rho(\mathbb{M}) = \frac{1}{2}\left(\frac{\alpha}{\alpha+\gamma} + \sqrt{\frac{\alpha^2}{(\alpha+\gamma)^2} + 4\frac{\alpha}{\alpha+\gamma}\frac{\kappa\rho N f(1)}{\beta\rho N + \mu}}\right) \tag{S9}$$

The Jury conditions state that $\rho(\mathbb{M}) < 1$ if and only if trace$(\mathbb{M}) <_* 1 + \det(\mathbb{M}) <_{**} 2$. The second $(**)$ Jury inequality is easily satisfied as $\det(\mathbb{M}) < 0$. The first $(*)$ is satisfied if

$$\mathcal{R}_0^* := \frac{\alpha\beta\rho N f(1)}{\gamma(\beta\rho N + \mu)} < 1.$$

This condition can also be found by solving $\rho(\mathbb{M}) < 1$ directly.

$\square$

When $\mathcal{R}_0^* < 1$, the branching process is subcritical, and the disease will die out with probability 1. If the disease is supercritical $\mathcal{R}_0^* > 1$, then there are unique fixed points q_1, and q_2 such that the probability of ultimate disease extinction is $q_i^{i_1} q_2^{i_2}$ given $I(0) = i_1$ and $E(0) = i_2$.

The fixed points are found by solving

$$f_1(q_1, q_2) = \frac{\alpha q_1 q u_2 + \gamma}{\alpha + \gamma} = q_1 \tag{S10}$$

$$f_2(q_1, q_2) = \frac{\kappa\rho N f(1) q_1 + \kappa\rho N(1 - f(1)) + \mu}{\beta\rho N + \mu} = q_2 \tag{S11}$$

This admits the following solution

$$q_1 = \frac{\gamma(\kappa\rho N + \mu)}{\alpha\kappa\rho N f(1)} = \frac{1}{\mathcal{R}_0^*} \tag{S12}$$

$$\begin{aligned} q_2 &= \frac{\alpha\kappa\rho N(1 - f(1)) + \kappa\rho N\gamma + \mu(\alpha + \gamma)}{\alpha(\kappa\rho N + \mu)} \\ &= \left(\frac{1}{\mathcal{R}_0^*} \cdot f(1) + (1 - f(1))\right) \frac{\kappa\rho N}{\kappa\rho N + \mu} + \frac{\mu}{\kappa\rho N + \mu} \end{aligned} \tag{S13}$$

These fixed points have epidemiological interpretations. An infectious individual successfully transmits an infection with probability $1/\mathcal{R}_0^*$. A pathogen either dies with probability $\mu/(\kappa\rho N + \mu)$ or is picked up with probability $\kappa\rho N/(\kappa\rho N + \mu)$. If the pathogen is picked up, it either does not cause disease with probability $1 - f(1)$ or it does with probability $f(1)$. If it causes disease, the probability of successfully transmitting an infection is $1/\mathcal{R}_0^*$.

Table S2: Transition events and rates for the stochastic analog of the environmentally mediated infectious disease transmission model with dose–response relationship given in Eqs. (3). Because ρW^* will typically not be an integer, we assign an integer number in the following way: $\lfloor \rho W^* \rfloor$ with probability $1-(\rho W^* - \lfloor \rho W^* \rfloor)$ and $\lceil \rho W^* \rceil$ with probability $\rho W^* - \lfloor \rho W^* \rfloor$.

Description	Transition	Rate
Pathogen pick up by S that causes disease	$(S, I, R, W^*) \to (S-1, I+1, R, W^* - \lfloor \rho W^* \rfloor)$	$\kappa S f(\min(\lfloor \rho W^* \rfloor, W^*)) \cdot (1-(\rho W^* - \lfloor \rho W^* \rfloor))$
Pathogen pick up by S that causes disease	$(S, I, R, W^*) \to (S-1, I+1, R, W^* - \lceil \rho W^* \rceil)$	$\kappa S f(\min(\lceil \rho W^* \rceil, W^*)) \cdot (\rho W^* - \lfloor \rho W^* \rfloor)$
Pathogen pick up by S that does not cause disease	$(S, I, R, W^*) \to (S, I, R, W^* - \lfloor \rho W^* \rfloor)$	$\kappa S(1-f(\min(\lfloor \rho W^* \rfloor, W^*)) \cdot (1-(\rho W^* - \lfloor \rho W^* \rfloor))$
Pathogen pick up by S that does not cause disease	$(S, I, R, W^*) \to (S, I, R, W^* - \lceil \rho W^* \rceil)$	$\kappa S(1-f(\min(\lceil \rho W^* \rceil, W^*)) \cdot (\rho W^* - \lfloor \rho W^* \rfloor)$
Pathogen pick up by I	$(S, I, R, W^*) \to (S, I, R, W^* - \lfloor \rho W^* \rfloor)$	$\kappa I \cdot (1-(\rho W^* - \lfloor \rho W^* \rfloor))$
Pathogen pick up by I	$(S, I, R, W^*) \to (S, I, R, W^* - \lceil \rho W^* \rceil)$	$\kappa I \cdot (\rho W^* - \lfloor \rho W^* \rfloor)$
Pathogen pick up by R	$(S, I, R, W^*) \to (S, I, R, W^* - \lfloor \rho W^* \rfloor)$	$\kappa W^* \cdot (1-(\rho W^* - \lfloor \rho W^* \rfloor))$
Pathogen pick up by R	$(S, I, R, W^*) \to (S, I, R, W^* - \lceil \rho W^* \rceil)$	$\kappa R \cdot (\rho W^* - \lfloor \rho W^* \rfloor)$
Recovery	$(S, I, R, W^*) \to (S, I-1, R+1, W^*)$	γI
Pathogen shedding	$(S, I, R, W^*) \to (S, I, R, W^*+1)$	αI
Pathogen decay	$(S, I, R, W^*) \to (S, I, R, W^*-1)$	μW^*

5. Global dynamics results

We extend Theorem 1 to include person-to-person transmission.

Corollary 1. *Let* $\Theta = \{(S, 0, 0, R, 0) : S + R = N\}$*. If* f *is concave down in Eqs. (S2), then, if* $\mathcal{R}_0 < 1$*,* Θ *is globally asymptotically stable.*

Proof. First, we note that $\Omega = \{(S, E, I, R, W) : 0 \le S \le N,\ 0 \le W \le N,\ 0 \le I \le N,\ 0 \le R \le N,\ 0 \le E \le \frac{\alpha N}{\xi}\}$ is a compact, invariant set for trajectories of Eqs. S2. In the notation of [S12], let $x = (E, I, W)$ be the disease compartments and $y = (S, R)$ the non-disease compartments. Then, we may write

$$\dot{x} = \mathcal{F} - \mathcal{V} \tag{S14}$$

where

$$\mathcal{F} = \begin{bmatrix} (\kappa f(\rho W) + \beta I/N)S \\ 0 \\ 0 \end{bmatrix}, \tag{S15}$$

$$\mathcal{V} = \begin{bmatrix} \sigma E \\ \gamma I - \sigma E \\ -\alpha I + \xi W \end{bmatrix}. \tag{S16}$$

Then we have new-infection and compartment transfer matrices

$$F = \begin{bmatrix} 0 & \beta & \kappa \rho N f'(0) \\ 0 & 0 & 0 \\ 0 & 0 & 0 \end{bmatrix}, \tag{S17}$$

$$V = \begin{bmatrix} \sigma & 0 & 0 \\ -\sigma & \gamma & 0 \\ 0 & -\alpha & \xi \end{bmatrix}. \tag{S18}$$

Then, the next generation matrix is $K = FV^{-1}$, and $\mathcal{R}_0$ is the spectral radius of K, namely

$$\mathcal{R}_0 = \frac{\alpha \kappa \rho N f'(0)}{\gamma \xi} + \frac{\beta}{\gamma}. \tag{S19}$$

Define

$$
\begin{aligned}
h(x,y) &= (F-V)x - \mathcal{F}(x,y) + \mathcal{V}(x,y) \\
&= \begin{bmatrix} \kappa\rho N f'(0)E - \kappa S f(\rho E) + \beta I(1-S/N) \\ 0 \\ 0 \end{bmatrix}.
\end{aligned} \tag{S20}
$$

Assume that f is concave down. Then $\rho E f'(0) \geq f(\rho E)$, and $h(x,y) \geq 0$.

The rest of the proof proceeds as in that of Theorem 1. □

6. Identifiability

Finally, we prove Theorem 2 using differential algebra techniques [S13, S14, S15].

Theorem 2. *The identifiable combinations of the model given in (Eqs (4)) given time series data of prevalence of infected individuals I are $\alpha\pi\kappa\rho$, ξ, $\gamma+\sigma$, and $\gamma\sigma$. If the time series of the environmental compartment W is also observed, then α is separately identifiable.*

Proof. First, we prove the theorem for prevalence data I. The model equations are

$$0 = \dot{S} + \pi\kappa\rho W S \tag{S21}$$

$$0 = \dot{E} - \pi\kappa\rho W S + \sigma E \tag{S22}$$

$$0 = \dot{I} - \sigma E + \gamma I \tag{S23}$$

$$0 = \dot{R} - \gamma I \tag{S24}$$

$$0 = \dot{W} - \alpha I + \xi W \tag{S25}$$

Eq. (S24) gives us no parametric information. Solving Eq. (S21) for W, $W = -\frac{\dot{S}}{\pi\kappa\rho S}$, the other three equations become

$$0 = \dot{E} + \dot{S} + \sigma E \tag{S26}$$

$$0 = \dot{I} - \sigma E + \gamma I \tag{S23}$$

$$0 = -\ddot{S}S + \dot{S}^2 - \alpha\pi\kappa\rho I S^2 - \xi \dot{S} S \tag{S27}$$

Then, solving the second equation for E, $E = \frac{\dot{I}+\gamma I}{\sigma}$, the other equations become

$$0 = \ddot{I} + (\gamma + \sigma)\dot{I} + \gamma\sigma I + \sigma\dot{S} \tag{S28}$$

$$0 = -\ddot{S}S + \dot{S}^2 - \alpha\pi\kappa\rho I S^2 - \xi\dot{S}S \tag{S27}$$

Using Ritt's pseudodivision [S15] and substituting $p_1 = \gamma$, $p_2 = \alpha\kappa\rho\pi$, $p_3 = \xi$, and $p_4 = \sigma$ we arrive at the following input–output equation:

$$\begin{aligned}
0 = &-4p_1^2p_4^3I^6 - 12p_1^2p_3p_4^2I^5\dot{I} - 12p_1^2p_4^2I^5\ddot{I} - p_1p_2^2p_4^3I^5 + -12p_1^2p_3^2p_4I^4\dot{I}^2 - 24p_1^2p_3p_4I^4\dot{I}\ddot{I} - p_1p_2p_4^2(3p_2p_3 + 2p_4)I^4\dot{I} \\
&- 12p_1^2p_4I^4\ddot{I}^2 - p_1p_4^2\left(3p_2^2 + 6p_2p_3 + 4p_4\right)I^4\ddot{I} - 2p_1(3p_2 + 2p_3)p_4^2I^4I^{(3)} - 4p_1p_4^2I^4I^{(4)} - 4p_1^2p_3^3I^3\dot{I}^3 - 12p_1^2p_3^2I^3\dot{I}^2\ddot{I} \\
&+ p_1p_4\left(-3p_2^2p_3^2 + 4p_4^2\right)I^3\dot{I}^2 - 12p_1^2p_3I^3\dot{I}\ddot{I}^2 - 2p_1p_3p_4\left(3p_2^2 + 6p_2p_3 + p_4\right)I^3\dot{I}\ddot{I} + 2p_1p_4\left(-6p_2p_3 - 4p_3^2 + 3p_4\right)I^3\dot{I}I^{(3)} \\
&- 8p_1p_3p_4I^3\dot{I}I^{(4)} - 4p_1^2I^3\ddot{I}^3 - p_1p_4\left(3p_2^2 + 12p_2p_3 - 3p_3^2 + 8p_4\right)I^3\ddot{I}^2 - 2p_1(6p_2 + p_3)p_4I^3\ddot{I}I^{(3)} - 8p_1p_4I^3\ddot{I}I^{(4)} \\
&- p_2^2p_4^2(p_2p_3 + p_4)I^3\ddot{I} + 3p_1p_4I^3(I^{(3)})^2 - p_2^2(p_2 + p_3)p_4^2I^3I^{(3)} - p_2^2p_4^2I^3I^{(4)} + p_1p_3\left(-p_2^2p_3^2 + 6p_2p_3p_4 + 6p_4^2\right)I^2\dot{I}^3 \\
&+ p_1\left(-3p_2^2p_3^2 + 2p_4\left(p_3^2 + 3p_4\right) - 6p_2\left(p_3^3 - 2p_3p_4\right)\right)I^2\dot{I}^2\ddot{I} - 2p_1p_3\left(3p_2p_3 + 2p_3^2 - 3p_4\right)I^2\dot{I}^2I^{(3)} - 4p_1p_3^2I^2\dot{I}^2I^{(4)} \\
&+ p_2^2p_4^2(p_2p_3 + p_4)I^2\dot{I}^2 + p_1\left(-3p_2^2p_3 + 3p_3^3 - 2p_3p_4 + 6p_2\left(-2p_3^2 + p_4\right)\right)I^2\dot{I}\ddot{I}^2 - 2p_1\left(6p_2p_3 + p_3^2 - 3p_4\right)I^2\dot{I}\ddot{I}I^{(3)} \\
&- 8p_1p_3I^2\dot{I}\ddot{I}I^{(4)}p_2p_4\left(-2p_2p_3p_4 - p_4^2 + p_2^2\left(-2p_3^2 + p_4\right)\right)I^2\dot{I}\ddot{I} + 3p_1p_3I^2\dot{I}(I^{(3)})^2 - p_2p_3p_4\left(2p_2^2 + 2p_2p_3 + p_4\right)I^2\dot{I}I^{(3)} \\
&- p_2p_4(2p_2p_3 + p_4)I^2\dot{I}I^{(4)} - p_1\left(p_2^2 + 6p_2p_3 - 3p_3^2 + 4p_4\right)I^2\ddot{I}^3 + 2p_1(-3p_2 + p_3)I^2\ddot{I}^2I^{(3)} - 4p_1I^2\ddot{I}^2I^{(4)} \\
&- p_4\left(2p_2^3p_3 + 2p_2p_3p_4 + p_4^2 + p_2^2\left(p_3^2 + 2p_4\right)\right)I^2\ddot{I}^2 + 3p_1I^2\ddot{I}(I^{(3)})^2 - 2(p_2 + p_3)p_4\left(p_2^2 + p_2p_3 + p_4\right)I^2\ddot{I}I^{(3)} \\
&- 2p_4\left(p_2^2 + p_2p_3 + p_4\right)I^2\ddot{I}I^{(4)} - (p_2 + p_3)^2p_4I^2(I^{(3)})^2 - 2(p_2 + p_3)p_4I^2I^{(3)}I^{(4)} - p_4I^2\left(I^{(4)}\right)^2 + p_1p_3^2(4p_2p_3 + 3p_4)I\dot{I}^4 \\
&+ 6p_1p_3(2p_2p_3 + p_4)I\dot{I}^3\ddot{I} + p_2p_4\left(2p_2^2p_3^2 + 3p_2p_3p_4 + p_4^2\right)I\dot{I}^3 + 3p_1(4p_2p_3 + p_4)I\dot{I}^2\ddot{I}^2 \\
&+ \left(3p_2p_3p_4^2 + 2p_4^3 + p_2^2p_4\left(p_3^2 + 3p_4\right) - p_2^3\left(p_3^3 - 4p_3p_4\right)\right)I\dot{I}^2\ddot{I} + \left(-p_2^3p_3^2 + 3p_2p_4^2 + 2p_3p_4^2 - p_2^2\left(p_3^3 - 2p_3p_4\right)\right)I\dot{I}^2I^{(3)} \\
&+ \left(-p_2^2p_3^2 + 2p_4^2\right)I\dot{I}^2I^{(4)} + 4p_1p_2I\dot{I}\ddot{I}^3 + \left(-p_2^2p_3^3 - 2p_2^3\left(p_3^2 - p_4\right) + p_2p_3^2p_4 + 2p_3p_4^2\right)I\dot{I}\ddot{I}^2 \\
&+ \left(-2p_2^3p_3 + p_2^2\left(-4p_3^2 + 2p_4\right) + p_4\left(p_3^2 + 3p_4\right) - 2p_2\left(p_3^3 - 2p_3p_4\right)\right)I\dot{I}\ddot{I}I^{(3)} + p_3\left(-2p_2^2 - 2p_2p_3 + p_4\right)I\dot{I}\ddot{I}I^{(4)} \\
&- (p_2 + p_3)\left(p_2p_3 + p_3^2 - 3p_4\right)I\dot{I}(I^{(3)})^2 + \left(-2p_2p_3 - 2p_3^2 + 3p_4\right)I\dot{I}I^{(3)}I^{(4)} - p_3I\dot{I}\left(I^{(4)}\right)^2 \\
&- \left(p_2^3p_3 + p_4\left(-p_3^2 + p_4\right) + p_2^2\left(p_3^2 + p_4\right) - p_2\left(p_3^3 - 2p_3p_4\right)\right)I\ddot{I}^3 + \left(-p_2^3 - 3p_2^2p_3 + p_3^3 + p_2\left(p_3^2 - 2p_4\right)\right)I\ddot{I}^2I^{(3)} \\
&- \left(p_2^2 + 2p_2p_3 - p_3^2 + 2p_4\right)I\ddot{I}^2I^{(4)} + \left(-p_2^2 + p_2p_3 + p_3^2 + p_4\right)I\ddot{I}(I^{(3)})^2 - 2p_2I\ddot{I}I^{(3)}I^{(4)} - I\ddot{I}\left(I^{(4)}\right)^2 + (p_2 + p_3)I(I^{(3)})^3 \\
&+ I(I^{(3)})^2I^{(4)} + p_1p_3^3\dot{I}^5 + 3p_1p_3^2\dot{I}^4\ddot{I} + \left(p_2^3p_3^3 + 2p_2^2p_3^2p_4 - p_4^3\right)\dot{I}^4 + 3p_1p_3\dot{I}^3\ddot{I}^2 \\
&+ p_3\left(3p_2^3p_3 + p_2p_3p_4 - 2p_4^2 + 2p_2^2\left(p_3^2 + 2p_4\right)\right)\dot{I}^3\ddot{I} + \left(2p_2^2p_3^2 + p_2p_3^3 + \left(p_3^2 - 3p_4\right)p_4\right)\dot{I}^3I^{(3)} + p_3(p_2p_3 + p_4)\dot{I}^3I^{(4)} \\
&+ p_1\dot{I}^2\ddot{I}^3 + \left(3p_2^3p_3 + 2p_2p_3p_4 + p_4\left(-2p_3^2 + p_4\right) + 2p_2^2\left(2p_3^2 + p_4\right)\right)\dot{I}^2\ddot{I}^2 + p_3\left(4p_2^2 + 2p_2p_3 + p_3^2 - 4p_4\right)\dot{I}^2\ddot{I}I^{(3)} \\
&+ \left(2p_2p_3 + p_3^2 + p_4\right)\dot{I}^2\ddot{I}I^{(4)} + \left(p_3^2 - 3p_4\right)\dot{I}^2(I^{(3)})^2 + p_3\dot{I}^2I^{(3)}I^{(4)} + (p_2 + p_3)\left(p_2^2 + p_2p_3 - p_3^2 + p_4\right)\dot{I}\ddot{I}^3 \\
&+ \left(2p_2^2 + p_2p_3 - 2p_3^2 + p_4\right)\dot{I}\ddot{I}^2I^{(3)} + (p_2 + p_3)\dot{I}\ddot{I}^2I^{(4)} - 2p_3\dot{I}\ddot{I}(I^{(3)})^2 + \dot{I}\ddot{I}I^{(3)}I^{(4)} - \dot{I}(I^{(3)})^3
\end{aligned} \tag{S29}$$

Because this equation is an input–output equation (i.e. a monic polynomial in the data I and its derivatives that is equivalent to the original model), the coefficients are identifiable, and thus it is clear that p_1, p_2, p_3, and p_4 can be individually be identified. Because the input–output equation may be written in terms of $p_1 = \alpha\kappa\rho\pi$, $p_2 = \xi$, $p_3 = \gamma + \sigma$, and $p_4 = \gamma\sigma$, their constituents are not individually identifiable. Because γ and σ can each take only one of two values, they are locally identifiable; it is sufficient two know which one is larger, for example, to separately identify them.

Finally, we show that α is separately identifiable when case I and environment data W are both available. Returning to the original equations, we solve Eq. (S23) for E.

$$0 = \dot{S} + \pi\kappa\rho W S \tag{S21}$$
$$0 = \ddot{I} + (\gamma + \sigma)\dot{I} + \gamma\sigma I - \sigma\pi\kappa\rho S W \tag{S30}$$
$$0 = \dot{W} - \alpha I + \xi W \tag{S25}$$

We then solve Eq. S30 for S.

$$\begin{aligned} 0 = {} & W I^{(3)} - \dot{W}\ddot{I} + (\gamma + \sigma) W \ddot{I} + \kappa\rho\pi W^2 \ddot{I} - (\gamma + \sigma)\dot{W}\dot{I} + (\gamma + \sigma)\kappa\rho\pi W^2 \dot{I} + \gamma\sigma W \dot{I} \\ & - \gamma\sigma \dot{W} I + \gamma\sigma\kappa\rho\pi W^2 I \end{aligned} \tag{S31}$$
$$0 = \dot{W} - \alpha I + \xi W \tag{S25}$$

These are the input–output equations for this model and data, and the coefficients are the identifiable combinations: α, $\kappa\pi\rho$, ξ, $\gamma + \sigma$, and $\gamma\sigma$.

□

References

[S1] Murphy B, Clements M, Madore H, Steinberg J, O'Donnell S, Betts R, et al. Dose Response of Cold-Adapted , Reassortant Influenza A/California /1O/78 Virus (H1N1) in Adult Volunteers. The Journal of Infectious Diseases. 1984;149(5):816.

[S2] Murphy BR, Clements ML, Tierney EL, Black RE, Stienberg J, Chanock RM. Dose response of influenza A/Washington/897/80 (H3N2) avian-human reassortant virus in adult volunteers. The Journal of Infectious Diseases. 1985;152(1):225–229.

[S3] Li S, Spicknall IH, Koopman JS, Eisenberg JNS. Dynamics and control of infections transmitted from person to person through the environment. American Journal of Epidemiology. 2009;170(2):257–265.

[S4] Ward RL, Bernstein DI, Young EC, Sherwood JR, Knowlton DR, Schiff GM. Human rotavirus studies in volunteers: determination of infectious dose and serological response to infection. The Journal of infectious diseases. 1986;154(5):871–80.

[S5] Hornick RB, Woodward TE, McCrumb FR, Snyder MJ, Dawkins AT, Bulkeley JT, et al. Study of induced typhoid fever in man. I. Evaluation of vaccine effectiveness. Transactions of the Association of American Physicians. 1966;79:361–7.

[S6] Hornick RB, Greisman SE, Woodward TE, DuPont HL, Dawkins AT, Snyder MJ. Typhoid Fever: Pathogenesis and Immunologic Control. The New England journal of medicine. 1970;283(13):686–691.

[S7] DuPont HL, Chappell CL, Sterling CR, Okhuysen PC, Rose JB, Jakubowski W. The Infectivity of Cryptosporidium parvum in Healthy Volunteers. New England Journal of Medicine. 1995;332(13):855–859.

[S8] DuPont HL, Hornick RB, Dawkins AT, Snyder MJ, Formal SB. The response of man to virulent Shigella flexneri 2a. The Journal of infectious diseases. 1969;119(3):296–299.

[S9] DuPont HL, Hornick RB, Snyder MJ, Libonati JP, Samuel BF, Gangarosa EJ. Immunity in shigellosis. II. Protection induced by oral live vaccine or primary infection. The Journal of Infectious Diseases. 1972;125(1):12–16.

[S10] Hornick RB, Music SI, Wenzel R, Cash R, Libonati JP, Snyder MJ, et al. The Broad Street pump revisited: response of volunteers to ingested cholera vibrios. Bulletin of the New York Academy of Medicine. 1971;47(10):1181–91.

[S11] Allen LJS, Lahodny GE. Extinction thresholds in deterministic and stochastic epidemic models. Journal of Biological Dynamics. 2012;6(2):590–611.

[S12] Shuai Z, van den Driessche P. Global stability of infectious disease models using Lyapunov funtions. SIAM Journal on Applied Mathematics. 2013;73(4):1513–1532.

[S13] Saccomani MP, Audoly S, Bellu G, D'Angio L. A new differential algebra algorithm to test identifiability of nonlinear systems with given initial conditions. Proceedings of the 40th IEEE Conference on Decision and Control. 2001;4:3108–3113.

[S14] Audoly S, Bellu G, D'Angiò L, Saccomani MP, Cobelli C. Global identifiability of nonlinear models of biological systems. IEEE Transactions on Biomedical Engineering. 2001;48(1):55–65.

[S15] Meshkat N, Anderson C, DiStefano JJ. Alternative to Ritt's pseudodivision for finding the input-output equations of multi-output models. Mathematical Biosciences. 2012;239(1):117–123.
